# Fibronectin contributes to notochord intercalation in the invertebrate chordate, *Ciona intestinalis*

**DOI:** 10.1186/s13227-016-0056-4

**Published:** 2016-08-31

**Authors:** Fernando Segade, Christina Cota, Amber Famiglietti, Anna Cha, Brad Davidson

**Affiliations:** 1 Department of Anatomy and Cell Biology, University of Pennsylvania School of Dental Medicine, Philadelphia, PA 19104 USA; 2 Department of Biology, Swarthmore College, 500 College Ave., Swarthmore, PA 19081 USA; 3 Section on Biological Chemistry, National Institute of Dental and Craniofacial Research, National Institutes of Health, Bethesda, MD 20892 USA; 4 Department of Systems Biology, Harvard Medical School, Boston, MA USA

**Keywords:** Chordate evolution, Tunicates, Extracellular matrix, Fibronectin, Notochord, Convergent extension

## Abstract

**Background:**

Genomic analysis has upended chordate phylogeny, placing the tunicates as the sister group to the vertebrates. This taxonomic rearrangement raises questions about the emergence of a tunicate/vertebrate ancestor.

**Results:**

Characterization of developmental genes uniquely shared by tunicates and vertebrates is one promising approach for deciphering developmental shifts underlying acquisition of novel, ancestral traits. The matrix glycoprotein Fibronectin (FN) has long been considered a vertebrate-specific gene, playing a major instructive role in vertebrate embryonic development. However, the recent computational prediction of an orthologous “vertebrate-like” *Fn* gene in the genome of a tunicate, *Ciona savignyi,* challenges this viewpoint suggesting that *Fn* may have arisen in the shared tunicate/vertebrate ancestor. Here we verify the presence of a tunicate *Fn* ortholog. Transgenic reporter analysis was used to characterize a *Ciona Fn* enhancer driving expression in the notochord. Targeted knockdown in the notochord lineage indicates that FN is required for proper convergent extension.

**Conclusions:**

These findings suggest that acquisition of *Fn* was associated with altered notochord morphogenesis in the vertebrate/tunicate ancestor.

**Electronic supplementary material:**

The online version of this article (doi:10.1186/s13227-016-0056-4) contains supplementary material, which is available to authorized users.

## Background

The chordate phylum consists of three major subphyla, cephalochordates, tunicates and vertebrates. Due to extensive morphological similarities, the cephalochordates were traditionally considered the closest sister group to the vertebrates. Comparative genomic analysis has reversed this arrangement, placing the cephalochordates at the base of the chordates and the tunicates and vertebrates as sister groups [1, 2]. This phylogenetic rearrangement raises a number of critical questions regarding chordate evolution. What novel, distinguishing traits defined the shared tunicate/vertebrate ancestor? Which traits were gained or lost during the evolution of distinct tunicate and vertebrate lineages? How did gene network modifications drive the emergence of these key transitional traits? Illuminating these fundamental aspects of chordate evolution represents a daunting challenge. Tunicate and vertebrate body plans had already diverged dramatically by the early Cambrian, obscuring the nature of their most recent common ancestor [3–5]. In the vertebrates, whole-genome duplications have greatly increased developmental gene network complexity. In the tunicates, acquisition of a cellulose tunic and adaptation to a sessile, filter-feeding life style are associated with extensive morphological modifications [6]. However, tunicate tadpole larvae maintain recognizable chordate features including a notochord and dorsal neural tube [7]. Thus, studies of tunicate embryonic development represent a promising avenue for exploring vertebrate origins.

Research on tunicate embryogenesis primarily focuses on the ascidian, *Ciona intestinalis*. *Ciona* embryos are translucent and constructed from extremely low cell numbers, permitting high-resolution analysis of morphogenesis [8]. The simple, highly condensed *Ciona* genome has facilitated detailed characterization of gene networks driving fate specification of progenitor lineages and how these specification networks are linked to morphogenetic effectors [9, 10]. In particular, substantial progress has been made in delineating gene regulatory networks underlying tunicate notochord, neural tube and heart morphogenesis [11, 12]. Recent studies have revealed rudimentary neural crest and placode lineages along the borders of the tunicate neural plate along with a set of cardiac/pharyngeal mesoderm progenitors orthologous to the vertebrate secondary heart field [13–17]. These studies suggest that defining developmental features previously considered to have emerged in the vertebrate lineage first arose in the tunicate/vertebrate ancestor.

The identification and characterization of genes uniquely shared by tunicate and vertebrate genomes represent a promising avenue for illuminating shared ancestral traits. Recent studies indicate that the gene encoding the key matrix glycoprotein Fibronectin (*FN*) may have arisen in the tunicate/vertebrate ancestor [18, 19]. *FNs* were long considered an exclusively vertebrate gene family characterized by a conserved arrangement of FN type 1, type 2 and type 3 domains [20]. Although some invertebrate genes contain one or more FN domains, none had been found that displayed the characteristic domain organization of vertebrate FN family members. However, a recent computational analysis of the *Ciona savignyi* genome predicted the presence of a *Fn*-*like* gene containing all three domains in a vertebrate-like arrangement (*Cs*-*Fn*) [18]. An incomplete segment of an *Fn*-*like* gene is also predicted to occur in the *C. intestinalis* genome, and a recent study documented expression of this gene in the developing notochord [21]. By contrast, even though a cephalochordate genome has now been extensively sequenced [22], no *Fn* orthologs were detected. Thus, *Fn* may represent a tunicate/vertebrate synapomorphy. Alternatively, computational predictions of cionid *Fn* genes may represent inaccurate fusions of two or more separate genes containing similar FN domains.

Fibronectin is a key component of the vertebrate extracellular matrix (ECM) with multiple, essential roles in embryogenesis [23]. Integrin receptors bind to FN, driving focal adhesion maturation and modulating proliferation, survival and migration. Integrin binding also induces conformational changes in FN that promote fibril formation [23]. This process, termed inside-out signaling, alters ECM properties to promote long-term changes in regional cell behavior [24]. FN contributes to gastrulation, axis elongation, germ layer specification, axial patterning and morphogenesis of mesodermal tissues, including the notochord and somites [25–29]. Although substantial progress has been made in delineating the molecular basis of FN-dependent signaling, the role of FN in notochord development and other morphogenetic processes has not been precisely delineated [30]. Additionally, little is known regarding the regulation of FN expression in the notochord.

In this paper, we confirm the presence of a functional *Fn* gene in the tunicate *C. intestinalis*. Through transgenic reporter analysis, we have identified a *Fn* notochord enhancer and begun to delineate specific binding sites required for enhancer function. We have also employed targeted knockdown to explore FN function, demonstrating that FN is required for intercalation of notochord precursor cells. We discuss the evolutionary implications of these results and their significance in regard to understanding the regulation and function of vertebrate *FN* during notochord morphogenesis.

## Results

### Cloning of the full-length *Ciona intestinalis Fn* cDNA

To determine whether the computationally predicted *Ciona savignyi Fn* accurately represents an expressed, coherent transcript, we focused on identifying and sequencing a full-length orthologous transcript from *C. intestinalis.* BLAST interrogation of the comprehensive *C. intestinalis* ghost database [31] identified a candidate 6809-bp gene model (KH.S417.6.v1.A.ND1-1). SMART 7 protein domain analysis indicated that KH.S417.6.v1.A.ND1-1 codes for a FN-like protein with multiple FN3 domains. However, the lack of a recognizable signal peptide at the N-terminus indicated that KH.S417.6.v1.A.ND1-1 did not represent the complete *C. intestinalis Fn* gene. We therefore implemented a PCR approach to acquire the full-length *Ci*-*Fn* cDNA. Maturation of *Ciona* mRNAs often involves *trans*-splicing of short RNA leader (SL) sequences resulting in diverse mRNAs with common 5′ end sequences [32]. We reasoned that trans-splicing of *Ci*-*Fn* might allow us to amplify the uncharacterized 5′ end using an upstream primer matching the characterized SL sequence, in combination with a downstream anchoring primer matching a sequence within the KH.S417.6.v1.A.ND1-1 gene. Using this approach, we successfully amplified and cloned a ~4.0-kb fragment using total cDNA synthesized from Stage 13 *C. intestinalis* embryo RNA (throughout this study, embryos were staged in accordance with [33]). Sequence analysis showed that the 3′ end of this 4-kb fragment overlaps with the 5′ end of KH.S417.6.v1.A.ND1-1, while the 5′ end contained the SL *trans*-spliced sequence. A tentative Met initiator codon (position 76–78) was followed by an open reading frame encoding a protein with a putative signal peptide. This 5′ sequence partially aligns with scaffold KhC9 position 3261026–3302435; however, the first 2345 base pairs do not show any alignment within the KH genome assembly. A BLAST search of the *C. intestinalis* EST database found that the 5′ end of our 4-kb fragment matches two ESTs (BW038621.1 and BW036600) from a blood cell cDNA library, further confirming that this sequence fragment derives from an expressed *C. intestinalis* transcript. BLAST searches against vertebrate protein databases found the most significant matches to Fibronectin (*e* value = *e*^−06^). Therefore, we concluded that the 4-kb PCR product corresponded to the uncharacterized 5′ region of gene model KH.S417.6.v1.A.ND1-1. We then cloned and sequenced the predicted KH.S417.6.v1.A.ND1-1 fragment from cDNA along with segments of overlapping cDNA that definitively link KH.S417.6.v1.A.ND1-1 and the 4-kb fragment, allowing us to assemble a complete *Ci*-*Fn* cDNA sequence (GenBank under Accession No. KX766380).

### *Ciona intestinalis Fn* mRNA and gene structure

The full-length *Ci*-*Fn* mRNA is 11,328-nt long and contains a 5′ untranslated region of 75 nt, an open reading frame of 11,094 nt and a 3′ untranslated region of 159 nt. BLAST searches of the *C. intestinalis* genome (Joint Genome Institute v2.0) with the UCSC Genome Browser showed that the *Ci*-*Fn* cDNA matches the reverse strand in chromosome 09q between positions 982,695–929,348, indicating that the *Ci*-*Fn* gene spans 53.347 kb of genomic sequence. By comparison, the human *FN* gene (*HsaFN1*), representative of vertebrate *FN1* genes, spans 75.7 kb and is transcribed into an 8.9-kb mRNA (splice isoform 1) [34]. *Ci*-*Fn* is a more compact gene (21 % of the gene codes for exons) than its vertebrate ortholog (only 13 % of the *HsaFN1* gene is exon coding), consistent with the relative compaction of the *C. intestinalis* genome in comparison with vertebrate genomes [35]. *Ci*-*Fn* consists of a minimum of 75 exons. There are two gaps in the available genome assembly and, therefore, exons 2, 3 and 26, and its neighboring introns could not be accurately mapped. All the intron splice sites follow the canonical GT/AG rule. Median exon length is 144 ± 42 bp (minimum 86 bp [exon 86]; maximum 393 bp [exon 42]) (see Additional file 1: Table 2), whereas median intron length is 523 ± 213 bp (minimum 245 bp [intron 11]; maximum 1347 bp [intron 74]) (see Additional file 2: Table 3). Introns interrupt the coding sequence in all three possible phases (see Additional file 1: Table 2). Exons flanked by phase 0 boundaries (where codons are not split between adjacent exons) are the most abundant (28 %), especially in the center of the sequence (see Additional file 1: Table 2).

### *Ciona* FN protein structure and architectural domains

**Fig. 1:**
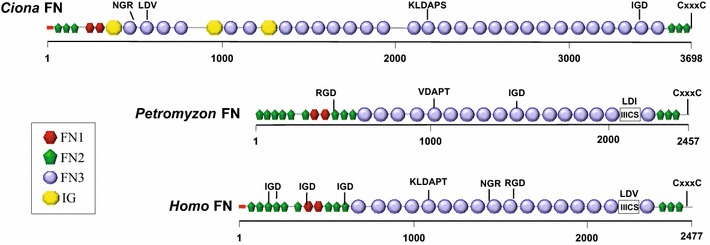
Comparison of the domain architecture of vertebrate and tunicate FN proteins. Protein domains in full-length *Ciona intestinalis* FN (3698 amino acids), human FN isoform 1 (accession number NP_001293061; 2477 amino acids) and lamprey (*Petromyzon*) FN (PMZ_0026015 [19]; fragment, 2457 amino acids) were identified with the SMART 7 domain prediction tool. FN and IG (immunoglobulin) domains as indicated in legend; *red box* signal peptide sequence; IIICS: type III connecting segment. Potential integrin-binding motifs (including IGD, NGR, KLDAP, DGR, LDV or RGD) and dimerization signal CxxxC are shown above the sequences

Conceptual translation of the open reading frame in the *Ci*-*Fn* cDNA generated a 3698-amino acid protein with an 18-amino acid signal peptide. The calculated molecular mass of the mature protein is 414 kDa, significantly higher than its human ortholog (262.6 kDa [isoform 1, UniProt P02751-1]). *Ci*-FN consists of 40 protein modules arranged in tandem arrays of three FN1 and two FN2 modules in its N-terminal region, a long tandem array of 29 FN3 modules interrupted by three Ig modules and a C-terminal repeat of three FN1 domains (Fig. 1; Additional file 2: Table 3). We have also identified a CTSTC sequence in the C-terminal region (position 3679–3683) that may represent a conserved CxxxC dimerization motif, also present in vertebrate FNs [36].

A comparison between tunicate and vertebrate FN proteins shows a common architecture, represented by a central core of FN3 repeats flanked by FN1 and FN2 modules. The number of repeated domains is variable throughout evolution and accounts for lineage-specific differences in protein size. Uniquely, tunicate FNs contain Ig domains interspersed within the FN3 tandem array (Fig. 1a, b). *Ci*-FN is also significantly longer than vertebrate FNs (e.g., *Hsa*-FN1 isoform 1 is 2477-amino acids long) and contains more FN domains (40 vs. 30 in *Hsa*-FN1), mostly due to a higher number of FN3 modules. *Ci*-FN contains a number of domains not represented in the predicted *Cs*-FN protein, including two N-terminal FN1, 11 FN3 modules, along with an additional FN1 and a dimerization motif at the C-terminus (see Fig. 1; Additional file 3: Table 4 and Additional file 4: Table 5). Whether the divergence between cionid FN transcripts reflects *bona fide* biological differences or it is the result of an incomplete computational prediction for *Cs*-*FN* is unclear.

The binding of integrin receptors to vertebrate FNs is primarily, although not exclusively, mediated by RGD binding sites located within the tenth FN3 module [36, 37]. However, this site is not conserved in lamprey FN (Fig. 1), suggesting that RGD-mediated integrin FN binding through this particular site arose in the jawed vertebrates [19]. *Ci*-FN contains no canonical RGD binding sites. Although an RGD site is predicted to reside in *Cs*-FN (module FN3-5, [18]), it is not located in an open loop suggesting a lack of functionality. However, *Ci*-FN does contain two potential integrin-binding sites, an LDV motif (in domain FN3-2) and a KLDAPT motif (in domain FN3-15a). In *Hsa*-FN similar motifs are bound by α4β1 and α4β7 integrins [37].

### Temporal expression of the *Ciona Fn* during embryogenesis

**Fig. 2:**
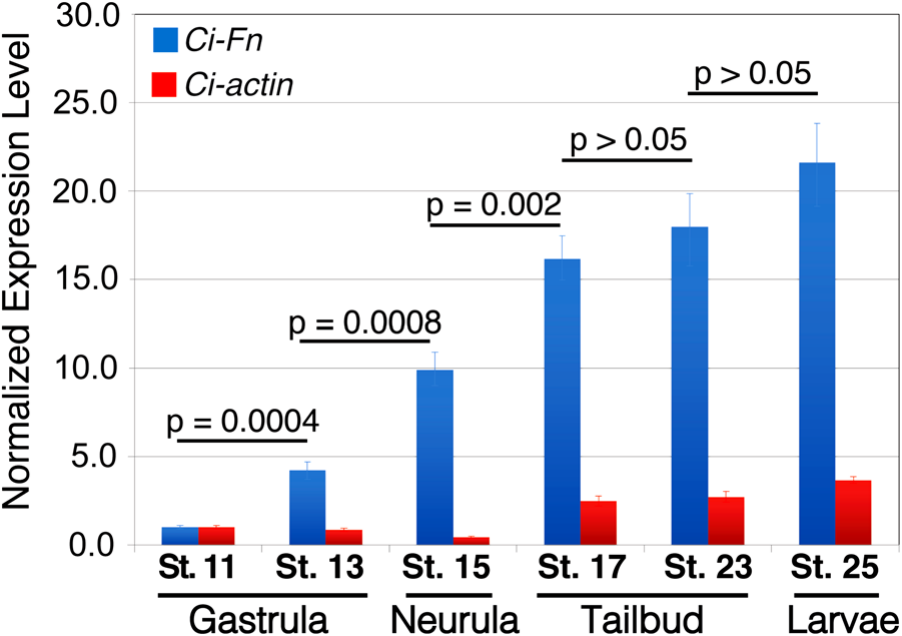
*Ci*-*Fn* mRNA accumulation throughout the development of Ciona embryos. Relative expression levels of *Ci*-*Fn* (*blue bars*) and *Ci*-*actin* (*red bars*) mRNA were obtained by quantitative PCR and normalized by parallel amplification of *Ciona 18S* target sequences and scaled in relation to minimal expression levels detected at Stage 11. *Bars* represent the mean ± SD for triplicate samples. *P* values are for the indicated pairwise comparisons by unpaired *t* test analysis

A recent study has established that *Ci*-*Fn* is strongly and specifically expressed in the notochord lineage [21] in mid-tailbud stage embryos (Stage 21), but the developmental timeline of expression has not been examined. Quantitative PCR was employed to ascertain relative levels of *Ci*-*Fn* mRNAs in staged samples ranging from early gastrula (Stage 11) to hatched larvae (Stage 25). Mean expression levels are shown in Fig. 2. The qPCR data indicate that *Ci*-*Fn* mRNA levels rise rapidly during gastrulation (Stages 11–13), showing a significant >fourfold increase by the end of gastrulation (*P* = 0.0004). Significant increases in expression continue until Stage 17 (early tailbud; *P* < 0.05) (Fig. 2), reaching a maximal 25-fold change by Stage 25 (swimming larva). The time frame of accelerated *Ci*-*Fn* expression spans a period of extensive notochord morphogenesis including intercalation, notochord cell elongation and lumen formation [21, 38, 39].

### Reporter analysis of a *Ciona Fn* minimal enhancer element

**Fig. 3:**
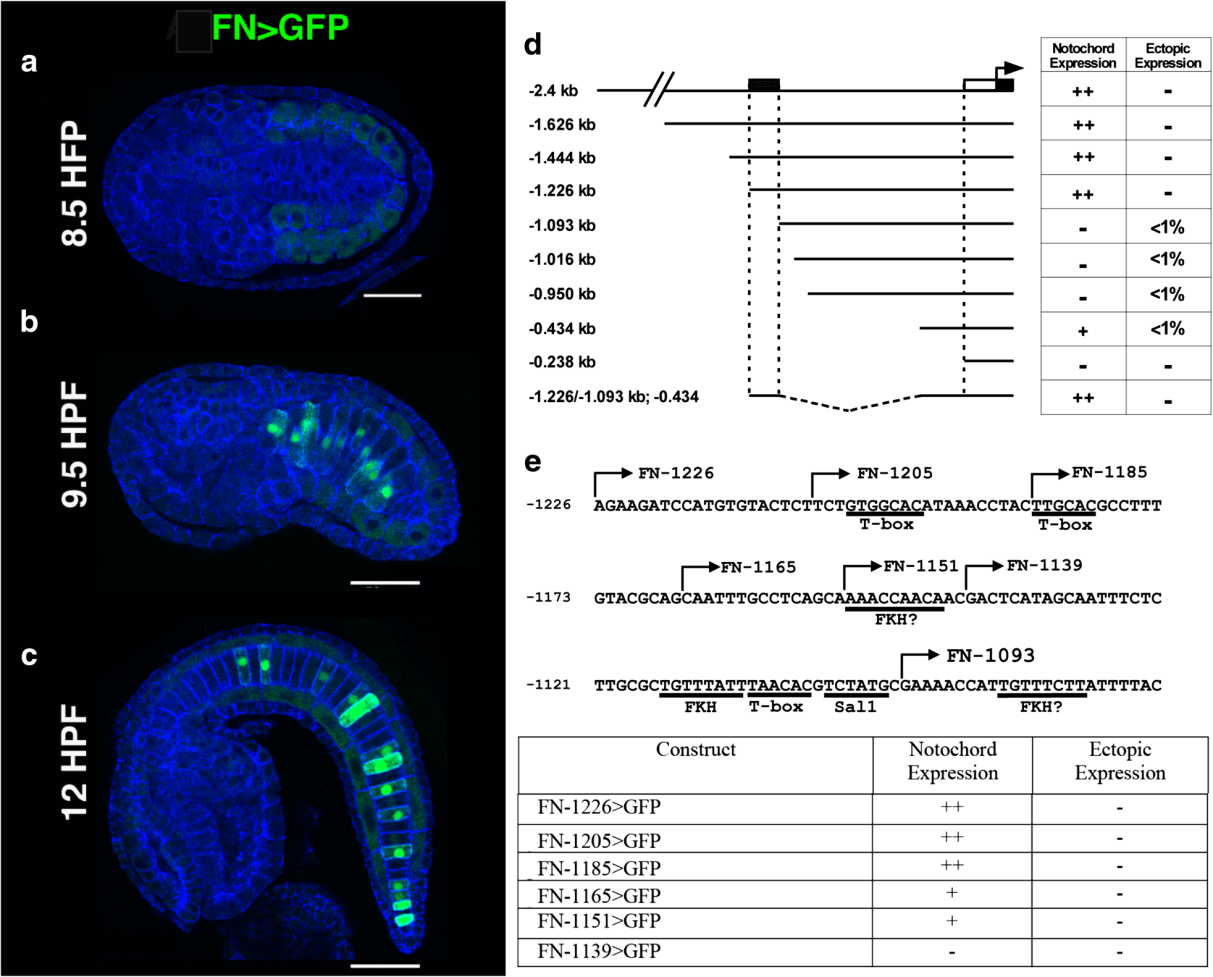
Reporter analysis of the *Ci*-*Fn* regulatory element. **a**–**c** Representative embryos displaying full-length Fn>*GFP* reporter expression at three different stages, *scale bars* = 50 μm. **a** At 8.5 h post-fertilization (HPF, Stage 17) reporter expression is not detected, background staining (*faint green*) is observed in developing muscle cells. **b** Mosaic FN reporter expression in the notochord lineage is initially detected at approximately 9.5 HPF (Stage 18/19). **c** *Ci*-*Fn* reporter expression in the notochord continues at later developmental stages as represented by a 12 HFP (Stage 21) embryo. **d** Schematic illustration of reporter constructs. The 5′ boundary relative to the initiator codon of the *Ci*-*Fn* sequence in each construct is identified by *numbers on the left*. *Table on the right* summarizes observed patterns of GFP expression. Relative abundances were estimated by comparing numerous transgenic embryos (>100 per construct). For ectopic expression, <1 % indicates the detection of GFP expression in non-notochord tissues in less than 1 in 100 embryos examined. *Stippled box* notochord enhancer region; *white rectangle*, core promoter; *black rectangle* 5′ untranslated region. **e** Map and reporter expression for deletions and mutations within the *Ci*-*Fn* minimal (−1226, −1093) enhancer region. Putative binding sites are underlined. *Arrows* indicate the 5′ ends of serial deletion constructs

We next began to investigate the regulation of *Ci*-*Fn* expression using transgenic reporter analysis. We amplified a 2.4-kb fragment spanning the intergenic region upstream of *Ci*-*Fn* (chr09q:982,618:985,000), excluding a 500-bp segment bordering the neighboring upstream gene (*Cionin*, LOC445737) to avoid the potential inclusion of competing regulatory elements. This 2.4-kb fragment was fused in-frame to a *Ciona*-optimized GFP coding sequence [40] to construct the parent Fn>*GFP* plasmid. Transgenic Fn>*GFP* embryos displayed strong, notochord-specific GFP expression (Fig. 3). Robust reporter expression was first detected in Stage 19 early tailbud embryos, ~9.5 h post-fertilization (HPF, Fig. 3b) and persisted throughout embryonic development and into larval stages (Fig. 3c; Additional file 5: Figure 1). Reporter expression was consistent and uniform in all stages examined. Interestingly, reporter expression was strongly enhanced in the most anterior pair of notochord cells in late larval samples (Additional file 5: Figure 1). This expression pattern may relate to notochord resorption during late larval settlement. Together, our results show that the 2.4-kb fragment of the *Ci*-*Fn* 5′ flanking region contains a functional promoter along with a *Ci*-*Fn* notochord enhancer.

To begin mapping functional elements in the 2.4-kb sequence, the *Ci*-*Fn* reporter was minimized through progressive 5′ deletions. The relative fluorescence signal and tissue specificity of each deletion construct were compared to those seen in embryos transfected with the parent Fn-2.4>*GFP* vector. Deletion from −2.4 to −1.226 kb did not discernably alter reporter expression. By contrast, further deletion to position −1.093 kb resulted in the complete loss of GFP expression in the notochord (Fig. 3d). These results indicate that essential elements of a notochord enhancer are present in the ~133-bp region spanning positions −1.226 to −1.093. We also ascertained that a reporter construct containing the proximal 434-bp fragment (Fn-43>*GFP*) showed minimal transcriptional activity, allowing us to use this region as a basal promoter for further analysis. We next investigated whether the 133-bp fragment (−1226/−1093) was sufficient to drive reporter expression when fused to the 434-bp basal promoter (Fn-1226/-1093:434>*GFP*). This minimal element drove notochord-specific reporter expression at levels of intensity similar to the full-length Fn-1226>*GFP* construct. To identify smaller enhancer fragments required for reporter expression, we examined the impact of incremental 5′ deletions on reporter expression (Fig. 3e). Removal of two distal 5′ 20-bp fragment (Fn-1205>*GFP,* Fn-1185>*GFP*) had no discernable impact on reporter expression. By contrast, deletion of an additional 20 base pairs led to reduced reporter activity (Fn-1165>*GFP)*. Further deletion to position −1151 had no additional impact. However, removal of 12 bp between positions −1151 and −1139 led to complete loss of reporter activity. These results suggest that transcription factor binding sites required for enhancer activity are present in the two small distal fragments (−1185:−1165 and 1151:1139). The gene regulatory network driving *Ciona* notochord gene expression has been extensively characterized. The T-box transcription factor Brachyury plays a primary, conserved role in initial specification of the notochord lineage during early cleavage. Multiple transcription factors include Ci-Tbx2/3, Ci-NFAT5, Ci-Lmx, Ci-Fos-a, Ci-Sall and Ci-Klf15 function downstream of Brachyury [21, 39]. We searched our 133-bp minimal enhancer for sequences matching binding site motifs for these characterized notochord transcriptions (Fig. 3e) and identified three consensus binding motifs for T-box transcription factors (TNNCAC; [41]) potentially mediating regulation by Brachyury or Tbx2/3. This element also contains a single candidate Sal1 binding site motif (TCTATG) [42]. No consensus sequences for NFAT, Fos, Lmx or KLF-15 were detected [43, 44]. The more distal functional fragment (−1185:−1165) contains one of the T-box consensus binding motifs. However, the more proximal fragment (−1151:−1139) does not contain any candidate TF binding motifs. We therefore employed the transcription factor prediction algorithm TFBIND to detect additional consensus motifs. The analysis revealed an imperfect match to a Fkh family transcription factor binding site (RARYAAAYA) in this 12-bp fragment along with two additional Fkh consensus sites at the proximal end of the 133-bp fragment. Together, this analysis has defined the boundaries of a functional notochord enhancer, giving us a platform to examine the contribution of individual binding motifs through targeted mutagenesis.

### Functional analysis of *Ciona Fn*

We next explored the functional role of FN through lineage-specific RNA interference (RNAi) (Bob Zeller, personal communication). RNAi constructs targeted two sequences, one in the 5′ UTR of the FN gene (FNHP57) and one exonic sequence in the middle of the FN gene (FNHP1998). BLAST comparisons ensured that there were no off-target matches for the selected target sequences. In these constructs, the RNAi hairpin is fused to a yellow fluorescent reporter protein (YFP) so that transgenic expression can be monitored. For our initial analysis, we employed an upstream *Forkhead* enhancer (Fkh) to drive hairpin expression in a broad domain that includes the notochord, endoderm and neural lineages. Fkh-driven expression of either hairpin (Fkh:FNHP1998 or Fkh:FNHP57) led to severe, pervasive developmental defects in comparison with control embryos electroporated with the Brac>*GFP* reporter (data not shown). Because Fkh:FNHP1998 showed greater penetrance, we focused further efforts on the FNHP1998 hairpin. We replaced the Fkh enhancer with the Brachyury enhancer to generate a construct capable of driving hairpin expression specifically in the notochord lineage (Brac>FNHP1998). To alleviate concerns about hairpin specificity, we also constructed a scrambled hairpin control construct. Transgenic embryos were reared to the late tailbud stage, following completion of notochord cell intercalation and elongation but prior to lumen formation. Embryonic phenotypes were grouped into three categories (Additional file 6: Figure 2A–D). Normal embryos had nearly perfect alignment of notochord cells and full tail extension. Embryos displaying minor notochord cell misalignments or slightly shortened tails were classified as moderately defective. Finally, embryos displaying nearly complete absence of notochord convergence and severely truncated tails were classified as severely defective. Embryos with pervasive embryonic defects unlikely to result from targeted disruption of notochord gene expression were scored separately and are not included in our analysis. It was clear from the control samples (Brac>*GFP*) that transgenesis alone led to a high incidence of mildly defective embryos (~35 %) (Additional file 6:Figure 2E). Thus, our analysis focused primarily on the incidence of severely defective phenotypes, which were rarely observed in the loading control (<10 %). Targeted expression of the FNHP1998 hairpin led to a robust and significant increase in the incidence of severely defective embryos (~80 %) in comparison with both Brac>*GFP* and scrambled hairpin controls (Additional file 6: Figure 2E). In the scrambled hairpin samples (Brac>ScFNHP1998), there was a significant increase in the incidence of mildly defective embryos indicating an off-target effect or general toxicity. However, the scrambled hairpin did not significantly impact the incidence of severely defective embryos. Taken together, these data suggest that FN function is required for proper notochord morphogenesis.

**Fig. 4:**
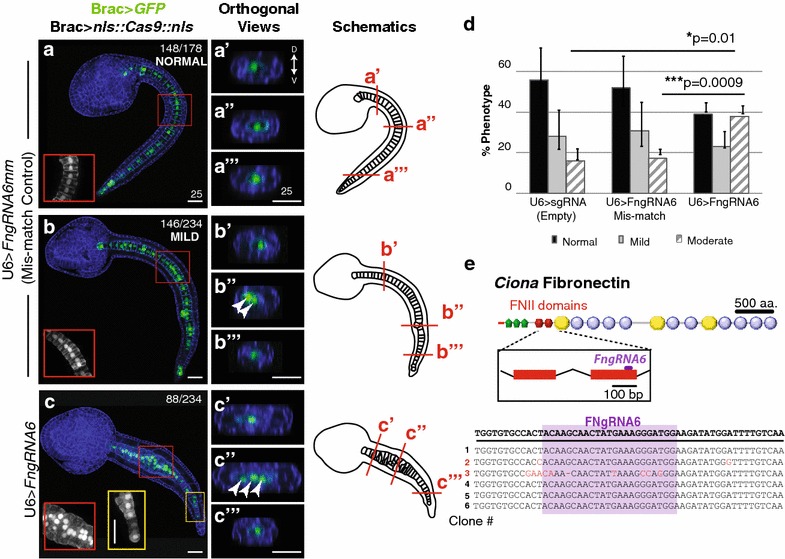
*Ciona* Fibronectin is necessary for intercalation of notochord cells during convergent extension. **a–c**′′′ Representative micrographs showing lateral projections and accompanying 2-μm orthogonal sections of notochord cells in late tailbud embryos co-transfected as labeled. *Red* and *yellow boxes* indicate location of *insets*. Approximate locations of orthogonal sections (**a**′**–c**′′′) are indicated in the associated schematics. Misaligned cells are indicated in orthogonal sections (*arrows*). Phalloidin (*blue*). Note that no reduction in notochord cell numbers occurred in these samples. In all 15 U6>FN6sgRNA embryos with bilateral incorporation of the Bra>GFP, 40 notochord cells were present. **d** Graphical summary of CRISPR/CAS9 data. Data were obtained from three independent trials, *n*>29/trial. *Error bars* represent the S.E.M. *P* values for moderately defective phenotypes in U6>sgRNA (empty) and U6>*FN6sgRNA* mismatch (controls) versus U6>*FN6sgRNA* are indicated. *P* = 0.882 for U6>*sgRNA mild* versus U6>*FNgRNA6* *mm mild.* Significance was determined using a two-tailed unpaired *t* test. **e** Approximate location of gRNA target sequence in FNII domain. Alignment of *FN* alleles cloned from pooled embryos electroporated with Brac>*nls::Cas9::nls* and U6>*FN6gRNA*. *Scale bars* 25 μm. Embryos oriented anterior to the *left*

**Fig. 5:**
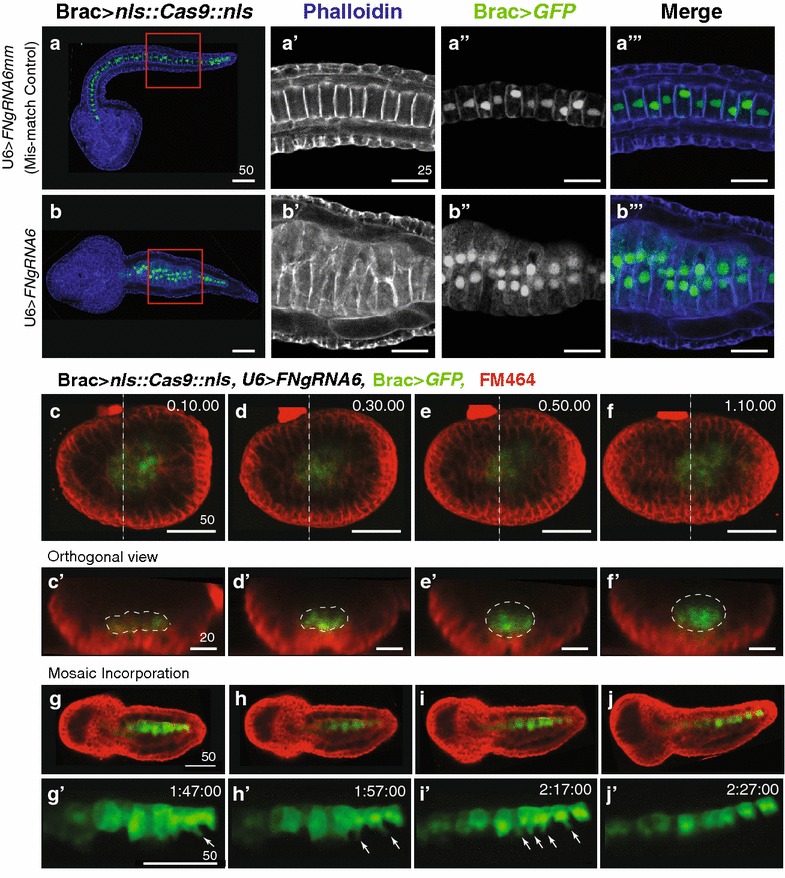
*Ci*-*Fn* knockdown specifically blocks the ability of medial protrusions to drive productive notochord intercalation. **a**, **b**′′′ Representative micrographs showing sections of phalloidin-stained (*blue*) late tailbud embryos co-transfected as labeled. *Red* and *boxes* (**a**, **b**) indicate location of zoomed in images (**a**′, **b**′′′). **a**′, **b** Representative section displaying typical medial lateral polarity of phalloidin enriched protrusions in non-intercalating cells. **a**′′, **b**′′ Representative section displaying typical medial localization of nuclei in non-intercalating cells. **c**, **j**′ Still images from movies of embryos co-transfected as labeled. **c**–**f**′ Representative embryo with bilateral incorporation of Brac>*GFP* showing invagination of notochord cells in 4-um orthogonal sections (*white outlines*, **c**′–**f**′) taken from Supplemental Movie 1. *Dashed lines* in (**c**–**f**) indicate location of orthogonal images (**c**′–**f**′). **g**–**j**′ Representative embryo with unilateral incorporation of Brac>*GFP* showing medial protrusions of notochord cells (*arrows*), taken from Supplemental Movie 5. Images represent 10- or 20-min intervals, time stamp in *upper right corner*. *Scale bars* in μm. Embryos oriented anterior to the *left*

We next employed CRISPR-Cas9 system for targeted *Fn* knockdown in the notochord lineage (Fig. 4). A guide RNA targeting the genomic sequence encoding the second FNII repeat was cloned into the previously characterized *Ciona* U6>*sgRNA(F* + *E)* template vector (U6>*FNgRNA6*; [45], Fig. 4e). To permit notochord lineage-specific knockdown, we placed *Cas9* under the control of the well-characterized *Brachyury* promoter (Brac>*nls::Cas9::nls*; [45, 46]). Previous work has demonstrated that single nucleotide substitutions in *Ciona* gRNA sequences prevent targeted knockdown [47]. We therefore employed single mismatch sgRNA (U6>*FNgRNA6* *mm)* as a stringent control. Each sgRNA was co-electroporated with Brac>*nls::Cas9::nls* and Brac>*GFP*. In general, disruptions in notochord morphology associated with CRISPR knockdown were less extreme than those observed in RNAi knockdown, ranging from normal to moderately defective. We therefore placed some embryos in a distinct “mildly defective” category indicating overall normal notochord morphology with scattered instances of abnormal cell behavior (Fig. 4b). In the majority of control embryos co-electroporated with either the template sgRNA targeting construct or sgRNA mismatch construct (Brac>*nls::Cas9::nls* + empty U6>*sgRNA vector or* U6>*FNgRNA6* *mm)* notochord development proceeded normally generating full tail extension and the typical single column alignment of notochord precursor cells (Fig. 4a). Although some control embryos displayed either mild or moderately defective notochord phenotypes, there was no significant difference between mismatch and empty vector controls (Figs. 4d, 5a, b″). By contrast, co-transfection with U6>*FNgRNA6* led to a robust and significant increase in the proportion of embryos displaying moderate defects in notochord morphology (Fig. 4c–c′′′, d). These moderately defective embryos were characterized by localized thickenings in which groups of notochord cells failed to properly intercalate (Fig. 4c; red inset). Cross sections clearly illustrate the single column characteristic of normal notochord morphology versus the multiple columns indicating localized disruptions in intercalation (compare Fig. 4a′–a′′′ to c′–c′′′). These defects did not arise from changes in proliferation, as the total number of notochord cells remained constant (see Fig. 4). The localized nature of the defects may reflect incomplete penetrance of CRISPR knockdown [50, 52], impacting specific lineages of transfected cells. Interestingly, the intercalation defects were consistently more severe in the anterior medial regions of the notochord (Fig. 4c–c′′′). Indeed, posterior notochord cells were often able to fully intercalate (Fig. 4c; yellow inset). In *Ciona*, the posterior-most notochord cells are derived from a separate, secondary lineage and these results suggest that targeted FN knockdown differentially impacts the primary versus secondary notochord cell populations. Alternatively, the general tapering of the tail may permit relatively normal convergence of posterior cells despite initial defects in intercalation. To confirm CRISPR-mediated *FN* mutagenesis, we amplified and sequenced the presumed CRISPR target region. In transgenic embryonic samples, mutations specific to the targeted region occurred in 17 % (1/6) of exonic sequences, including a nucleotide deletion predicted to alter the reading frame and produce a severely truncated FN protein (Fig. 4e). Taken together these results indicated that FN is required for notochord cell intercalation.

Notochord cell intercalation is driven by medio-lateral protrusive activity and relies on an intact sheath of matrix proteins [48, 49]. In phalloidin-stained U6>*FNgRNA6* samples, defective cells displayed medial–laterally enriched protrusions (Fig. 5b, b′). These cells also displayed medial localization of their nuclei (Fig. 5b′′, b′′′). These results suggest that FN is not required for medio-lateral polarization. It was also evident that defective notochord cells did not protrude into neighboring tissues, indicating that FN knockdown did not disrupt the integrity of the notochord sheath (Figs. 4c, 5b). Furthermore, defective cells did not exhibit protrusions along the notochord boundary (Figs. 4c, 5b). Thus, it appears that FN does not disrupt “boundary capture”-mediated inhibition of protrusive activity, a process that has been characterized in both vertebrate and tunicate embryos [48–51]. To more precisely evaluate the impact of FN knockdown on notochord morphogenesis, we imaged Brac>*GFP*-labeled notochord lineage cells in live embryos co-electroporated with U6>*FNgRNA6* and *Brac*>*nls::Cas9::nls*. GFP expression became detectable in late neurula stage embryos at which point the notochord rudiment formed a single cell-layered sheet characteristic of Stage 1 notochord morphogenesis (Fig. 5c, c′; Additional file 7: Movie 1) [38]. As embryos completed neurulation, this sheet invaginated to form the multi-layered rod characteristic of Stage II notochord morphogenesis [38] (Fig. 5c–f′; Additional file 8: Movie 2). In early tailbud stage embryos, notochord cells were clearly mobile, extending protrusions and jostling in relation to their neighbors (Additional file 9: Movie 3). However, as seen in fixed samples, subsets of notochord cells failed to intercalate. While some labeled cells extended in between opposing cells, defective cells appeared to form a stable bilayer, flattening out against opposing cells (Additional file 10: Movie 4). It was also clear that defective cells did not migrate into neighboring tissues, as seen when notochord sheath integrity is disrupted [48]. In some embryos, Brac>*GFP* incorporation was restricted to one side of the embryo (Fig. 5g–j′; Additional file 11: Movies and Additional file 12: Movie 6). In these samples, it was clear that defective cells are capable of initiating intercalatory behaviors, extending narrow medial protrusions that partially penetrate between opposing cells (arrows, Fig. 5g′–i′). However, these protrusions fail to progress and eventually retracted (Fig. 5j′). Taken together, analysis of live and fixed samples strongly suggests that *Fn* knockdown does not disrupt medio-lateral polarity or the notochord sheath. Instead, it appears that FN is required for medial protrusions to productively penetrate between opposing cells.

## Discussion

Our results confirm the presence of a *C. intestinalis Fn* ortholog, indicating that this critical developmental matrix protein first arose in the last common tunicate/vertebrate ancestor. Reporter studies of *Ciona Fn* regulation identified a 133-bp minimal regulatory element, and preliminary deletion analysis has identified two small (12 and 20 bp) functionally required distal fragments. Functional studies suggest that intercalating notochord cells require FN binding to effectively penetrate between opposing cells. A similar function for FN has been characterized in relation to vertebrate notochord intercalation [52]. The comparative and evolutionary implications of these results are addressed in the following sections.

### Insights into the emergence of Fibronectin within the chordates

Phylogenetic positioning of tunicate *Fn* genes in relation to vertebrate orthologs remains in flux. The fully sequenced *C. intestinalis Fn* gene shows a characteristic “vertebrate-like” arrangement of three distinct FN domain types. This arrangement has not been described in any other invertebrate protein [18]. While FN type 3 domains are pervasive, occurring in a wide range of metazoan proteins, FN type 1 domains have only been detected in deuterostome proteins and FN type 2 domains are restricted to chordate proteins [53]. The predicted *Cs*-*Fn* gene was initially characterized as a true ortholog to vertebrate family members [18]. However, a recent analysis has reassessed this classification, designating tunicate *Fn* genes (including *Cs*-*Fn* and a partially annotated larvacean *Fn* ortholog) as “*Fn*-*like,*” distinct from “true” vertebrate *Fn* genes [19]. Unquestionably, vertebrate *Fn* genes share a unique, highly conserved domain architecture that is not represented in tunicate *Fn* family members. In particular, tunicate *Fn* genes encode a lower number of FN1 domains at the N-terminus and contain Ig domains not present in vertebrate *Fn* genes. However, from an evolutionary standpoint, designation of vertebrate family members as “true” *Fn* genes is arbitrary. A more evolutionarily accurate, unbiased terminology is warranted, with gene designations that reflect hypothesized phylogenetic relationships. Considering that the tunicates and vertebrates are sister taxa and also taking into account the lack of any *Fn* or *Fn*-*like* genes in the cephalochordates (represented by the *Branchiostoma* genome [22]) or in the non-chordate deuterostomes [19], it is most parsimonious to assume that the tunicate and vertebrate FN orthologs were derived from a shared ancestral tunicate/vertebrate *Fn* gene. Otherwise, one must posit parallel acquisition of FN2 domains and the convergent ordering of all three domains in each clade. Additionally, loss of a *Fn* gene in *Branchiostoma* is relatively unlikely considering the overall conservative character of the *Branchiostoma* genome [22]. Thus, we propose that *Fn* was acquired in the vertebrate/tunicate ancestor in association with novel developmental or physiological roles.

It will be extremely difficult to determine whether the presumptive, ancestral chordate Fn protein more closely resembled tunicate or vertebrate derivatives. In particular, the lack of an RGD motif and the presence of Ig domains in *Ciona* FN may represent ancestral features or may have been derived within the tunicates. In vertebrates, the RGD motif mediates most of the protein integrin-binding activity and is required for FN fibrillogenesis [36]. However, *Ciona* FN does contain non-RGD motifs similar to characterized vertebrate motifs bound by integrin [37]. Functional studies of *Ciona* integrin/FN binding are required to clarify whether *Ciona* FN mediates cell–matrix interactions though these motifs. In regard to the Ig domains in *Ciona* FN, their presence in the N-terminal half of the molecule may relate to fibrillogenesis and ECM interactions associated with this region [36]. FNIII and Ig domains are notably similar in three-dimensional structure [54, 55], and they are present in tandem arrays in a number of vertebrate proteins, most notably titin [56]. In titin, Ig and FNIII domains respond in a highly similar manner to mechanical forces and synergistically contribute to protein elasticity [57]. Thus, Ig domains may alter the flexibility of the N-terminal region of *Ciona* FN, thereby modulating FN fibrillogenesis. The presence of an Ig domain in a partial *Oikoipleura Fn* gene model [19] indicates that this feature was present in the ancestral tunicate *Fn* gene. Characterization of full-length *Fn* genes from additional tunicates will clarify whether Ig domains or the lack of an RGD motif is ancestral to the tunicates or derived within *Ciona*.

### Regulation of *Ci*-*Fn* within the *Ciona* notochord gene regulatory network

In *C. intestinalis*, the T-box transcription factor *Brachyury* is the primary regulator of notochord specification and morphogenesis [39, 58]. Extensive characterization of the *Ciona* notochord GRN has identified a comprehensive set of Brachyury target genes including the transcription factors *Ci*-*Tbx2/3, Ci*-*NFAT5, Ci*-*AFF, Ci*-*Fos*-*a, Ci*-*Sal* and *Ci*-*Klf15* [39, 59]. Temporal expression of these downstream notochord TFs are associated with distinct morphogenetic stages including intercalation, formation of the notochordal sheath and lumen formation [21, 39]. Microarray data suggest that *Ci*-*Tbx2/3* regulates downstream notochord genes including *Ci*-*Fn* [21]. Although the vertebrate notochord GRN remains incompletely characterized, *Brachyury* is known to play a key, presumably conserved role in notochord specification [60, 61]. Numerous Brachyury downstream targets have been identified including genes encoding ECM components, integrin receptors, connective tissue growth factor and other morphogenetic factors [61, 62]. The minimal *Fn* enhancer element identified in this study will facilitate the cis-mutational analysis of candidate binding sites along with the functional characterization of candidate trans-factors required to precisely define *Ci*-*Fn* regulation. Further insights into *Ciona* FN regulation will illuminate the evolution of regulatory circuits for *Fn* and other peripheral effectors in chordate notochord networks.

### Insights regarding the contribution of fibronectin to notochord morphogenesis

Our targeted knockdown data demonstrate that *Ciona* FN is required for intercalation of notochord progenitors (Stage III of morphogenesis according to [38]). Knockdown of *Fn* does not disrupt oriented cell divisions required to generate an initial monolayered sheet of notochord precursors (Stage I) or subsequent invagination to form a thick rod (Stage II) [38, 63]. Subsequent morphogenesis, including expansion along the anterior/posterior axis (Stage IV); luminal vacuole formation (Stage V); and merger to form a hollow rod (Stage VI) [38], is also disrupted, but it is not clear whether this is due to a general arrest in notochord development or to a specific requirement for FN during later stages. The precise contribution of FN to *Ciona* notochord intercalation remains ambiguous. Previous work has demonstrated that intercalation requires both planar cell polarity (PCP) and interactions with the notochord sheath matrix. Perturbations that disrupt PCP, including mutation of the *Ciona prickle* ortholog (*aim*) or manipulations of Wnt5 signaling, lead to severe defects in notochord cell intercalation [49, 64]. However, PCP defective cells are able to partially converge, forming two rows. Partial convergence appears to be mediated by inhibition of protrusive activity along the notochord boundary (boundary capture) leading to a medial–lateral protrusive bias. Internally secreted laminin, observed at the interface between converging notochord cells in *aim* mutants, may also contribute to protrusive polarity. Loss of sheath integrity, resulting from mutation of the sole *Ciona α*-l*aminin* ortholog (*chongmague*), also disrupts intercalation [48]. In *chongmague* mutants, notochord cells protrude and migrate into neighboring tissues. Additionally, boundary capture is disrupted. Knockdown of *Fn* does not appear to disrupt sheath integrity. In U6>*FNgRNA6* embryos, the notochord rudiment remains coherent and defective cells do not escape into adjoining tissues (Figs. 4, 5). Additionally, defective cells do not exhibit protrusive activity along the boundary, indicating that FN is not required for boundary capture. The impact of *Fn* knockdown on PCP is difficult to assess. Defective *Fn* knockdown cells are able to produce extended medial protrusions (Fig. 5). However, these polarized protrusions may result from boundary-mediated polarization or internal laminin rather than functional PCP. Indeed, the partial convergence of FN knockdown cells to form two opposing rows is similar to the *aim* mutant phenotype [49]. *Fn* knockdown may also impact cell–cell adhesion as characterized in vertebrate embryos. Following functional perturbation of *FN* or the FN-binding Int*α5*ß1 heterodimer, intercalating vertebrate cells remain protrusive, exhibit boundary capture and participate in partial convergence [52, 65]. Thus, disruption of FN binding leads to similar phenotypes in vertebrate and *Ciona* embryos. In vertebrate embryos, it has been shown that FN adhesion contributes to intercalation indirectly by promoting the formation of cell–cell adhesions [52]. Without proper cell–cell adhesion, medial protruding cells are not able to effectively pull on their neighbors and complete intercalation. However, in vivo perturbations of FN/integrin function in vertebrates are difficult to interpret due to defects in gastrulation that may also impact notochord morphogenesis [28]. Cellular simplicity and lineage-specific knockdown make *Ciona* a valuable model for elucidating the precise contribution of FN to convergent extension.

### Fibronectin and emergent properties of the tunicate/vertebrate ancestor

The acquisition of FN in the tunicate/vertebrate ancestor may have been associated with alterations in notochord structure, function or morphogenesis. In cephalochordates, the notochord functions as a contractile hydrostatic skeleton [66]. Amphioxus notochord cells express genes encoding muscle components and show characteristic features of both smooth and skeletal muscle, including centrally located nuclei and thick and thin filaments [67]. In contrast, tunicate and vertebrate notochord cells are non-contractile. Thus, FN may have initially been deployed in the tunicate/vertebrate ancestral notochord, helping to provide novel mechanical properties associated with a profound shift in notochord structure and function. Although structurally distinct, the cephalochordate notochord is also formed through intercalation and convergent extension. However, in cephalochordate embryos intercalation takes place after the notochord rudiment folds to form a rod two cell widths across. Thus, cells only intercalate with one opposing row [68]. By contrast, tunicate and vertebrate notochord morphogenesis involves intercalation of a broad plate with multiple cell rows (>10 rows in zebrafish, 5–10 rows in mice and 8 cell rows in *Ciona*) [69–73]. It is possible therefore that FN was initially deployed in association with a divergent mode of intercalation.

Alternatively, FN may have been acquired in association with a novel mode of gastrulation in the tunicate/vertebrate ancestor. In cephalochordate gastrulation, endomesodermal cells invaginate as a cup-like structure [77]. This may represent a basal chordate mode of gastrulation similar to the invagination of the archenteron in non-chordate deuterostomes. In vertebrates and tunicates, endomesoderm cells involute, crawling along the ectoderm as they internalize. FN plays a key role in guiding vertebrate mesoderm involution [74]. This may represent an ancestral function for FN, lost in tunicates due to drastic reductions in cell numbers and shifts in early patterning associated with rapid embryogenesis [75]. Alternatively, FN may still contribute to tunicate gastrulation. Published data indicate that *Ci*-*Fn* is specifically expressed in the developing notochord by the mid-tailbud stage, downstream of the notochord transcription factor Ci-Tbx2/3 [21]. Since *Ci*-*tbx2/3* is first expressed in the notochord at the end of neurulation, it is likely that *Ci*-*Fn* expression in notochord lineage initiates during early tailbud stages, mirroring the temporal expression of our reporter. However, our qPCR data indicate that *Ci*-*Fn* expression is up-regulated during gastrulation. Thus, initial *Ci*-*Fn* expression may relate to conserved functions associated with gastrulation. More broadly, changes in gastrulation and the associated use of FN may have accompanied shifts in early patterning and the emergence of a novel tunicate/vertebrate ancestral body plan. Testing of these highly speculative hypotheses will require more comprehensive *Ci*-*Fn* in situ expression data, early knockdown of *Ci*-*Fn* in early mesodermal and endodermal lineages, investigation of FN expression and function in additional tunicates and in depth comparisons of gastrulation and notochord morphogenesis throughout the chordates.

## Conclusions

Our findings strongly suggest that Fibronectin represents a tunicate/vertebrate synapomorphy. In tunicates, *Fn* is robustly expressed in the notochord and targeted loss of function assays indicates that FN facilitates effective convergent extension of intercalating notochord cells. Fibronectin may have been acquired in the tunicate/vertebrate ancestor in association with novel aspects of notochord morphogenesis or gastrulation. Further elucidation of FN function throughout the chordates should help illuminate the elusive nature of the last common ancestor shared by tunicates and vertebrates.

## Methods

### Materials and methods

#### Embryological techniques

Gravid *Ciona* adults were collected in San Diego County (M-Rep) and maintained at 18 °C under constant light to prevent spawning. It has recently become clear that *C. intestinalis* represent a species complex consisting of a number of cryptic species [76] and their taxonomic status remains in flux. According to re-classifications of *Ciona* subspecies [77], individuals harvested in San Diego likely represent *Ciona robusta,* also termed *C. intestinalis* type a. Embryos were fertilized dechorionated and electroporated according to standard techniques [46]. For electroporations, 100 μg of each construct was used to ensure highly penetrant incorporation.

#### RNA interference: hairpin construction

All hairpins were constructed using the *Ciona* RNA interference Instruction Manual version 1.1 recently developed by Robert W. Zeller (unpublished). Target sequences were BLASTed against transcriptome databases to ensure target specificity. Hairpins were first cloned into an assembly vector and then subcloned into a *Brachyury* expression vector. Hairpin construct folding was also checked to verify for no mismatches. Scrambled hairpin sequences with the same base composition as the targeting hairpins were generated using the SCRAMBLE tool at the GenScript Web site (https://www.genscript.com/ssl-bin/app/scramble).

#### Staining and confocal microscopy

Transgenic embryos were fixed overnight in 0.5 % paraformaldehyde in artificial seawater (Crystal Sea Marine Mix). For phalloidin staining embryos were rinsed twice in 1X PBS-BSA 1 % and 1X PBT followed by two PBS-BSA rinses. They were then incubated at RT in 1XPBS-BSA +1:250 Alexa Fluor 635 phalloidin (Invitrogen) for 2 h and rinsed twice in PBS-BSA. Embryos were mounted in glycerol and stored at 4 °C. Z-stack images (2-μm sections) were generated using a Leica SP5 confocal microscope.

#### RNA extraction and cDNA synthesis

Total RNA was purified from *Ciona* embryos using the acid-guanidinium thiocyanate-phenol–chloroform method with TRIzol reagent (Invitrogen). Yields were quantified spectrophotometrically. Single-stranded cDNA was synthesized from 5 μg of total RNA using 50 μg of random hexanucleotides as primers and 200 units of SuperScript III reverse transcriptase (Invitrogen) at 50 °C for 50 min, following the manufacturer’s instructions.

#### Isolation of *Ciona* genomic DNA

*Ciona intestinalis* genomic DNA was purified from freshly obtained sperm using the Qiagen DNeasy Blood and Tissue kit according to the manufacturer’s instructions.

#### Quantitative PCR

Reactions were performed with the Bio-Rad DyNAmo SYBR Green kit (Thermo Scientific). For each qPCR assay, 1 μl of a 1:100 cDNA dilution template (equivalent to the cDNA synthesized from 10 ng of total RNA) was used, in a final volume of 20 μl containing 1× SYBR Green master mix, and 250 nM primers. Amplification of *Ciona* 18S rRNA was used for normalization. After a denaturation step at 95 °C for 15 min, the amplification conditions were 45 cycles of denaturation at 94 °C for 20 s, annealing at 56 °C for 30 s and extension at 72 °C for 30 s. Readouts took place at the end of each extension step. A melting curve was generated at the end of the amplification to verify the specificity and integrity of the amplicons. Each reaction was done in triplicate, and reported values are the mean of each triplicate. For quantification, levels of *Ci*-*Fn* and *18S* were calculated from the threshold cycle number (Ct) during the exponential phase of the PCR amplification. The target *Ci*-*Fn* level was normalized by the Ct of *18S* as ΔCt = Ct(*Ci*-*FN*) − Ct(*18S*). For relative expression levels of *Ci*-*Fn* during development we normalized to level at Stage 11 as 1, and we calculated ΔΔCt as ΔΔCt = ΔCt(test stage) − Ct(Stage 11). Fold changes and standard deviations in target levels were calculated using the formula R = Mean efficiency^(−ΔΔC(t))^ [78]. Statistical significance of differences in expression levels was determined with the paired *t* test. The significance level was defined as *P* < 0.05.

#### Cloning of the full-length *Ciona Fn* cDNA

cDNA equivalent to 50 ng of total RNA was amplified with Platinum Taq DNA polymerase (Invitrogen) using 200 μM of the splice leader-specific primer SL-EcoF (TAAGGATCCGATTCTATTTGAATAAG) [32] and a downstream primer Ci-FN_Xho140R (TAACTCGAGCCTTCAATGACCTGCATAC), which matches a region 140-bp downstream of the 5′ end of the KH.S417.6.v1.A.ND1-1 gene model sequence in the *C. intestinalis* Ghost database [31]. The 4-kb amplification product was purified, TOPO-cloned into the pCR2.1 vector (Invitrogen) following the manufacturer’s recommendations and sequenced in its entirety (Genewiz, South Plainfield, NJ). Sequencing reads were analyzed and assembled with the MacVector software.

#### Construction of reporter vectors

A 2.5-kb fragment of the 5′ flanking region of the *Ci*-*Fn* gene immediately upstream of the putative initiator codon was amplified from *Ciona* genomic DNA (100 ng) using Platinum Taq DNA polymerase in the presence of primers pPromF (TATTGGAGAGGACAAAACGAGGAC) and pPromR (CATCTTGACTAACAAGAACCGCTT). The amplification products were purified, TOPO-cloned into the pCR2.1 and sequenced in their entirety. A collection of constructs containing variable lengths of the 5′ flanking region of the *Ci*-*Fn* gene was then generated by PCR amplification of the 2.5-kb 5′ flanking region. Forward primers were designed from sequences located at varying distances (see Results section) from the putative ATG initiator codon in *Ci*-*FN* and included the *Xba*I restriction endonuclease recognition site to facilitate cloning. Oligonucleotide FNpNotR, containing a *Not*I recognition sequence, was used as the common reverse primer with its 3′ end located at position +3 of the initiator ATG. Amplimers were then inserted into XbaI/NotI-digested promoter-less fragment of the *Ciona* expression vector Mesp>*GFP* [79] from which the *Mesp* promoter had been previously excised. Mutagenesis of transcription factor binding sites was carried out using the Fn-1226>*GFP* plasmid as template for the linear amplification of mutant strands with *Pfu*II Turbo DNA polymerase (Agilent Technologies) using the appropriate pair of complementary primers and 12 cycles of synthesis with extension time of 1 min/kb. The integrity of the constructs was verified by sequencing. Plasmids for electroporation were purified from bacterial cultures with the NucleoBond Xtra Midi EF/Midi Plus EF purification kit (Clontech).

#### Protein domain analysis

The Simple Modular Architecture Research Tool (SMART) version 7 (http://smart.embl-heidelberg.de/) was used to identify putative FN domains [80]. Additional domains were identified by BLAST comparison against Human FN1.

#### Molecular evolutionary analyses

FN1-encoding sequences from representative vertebrate species were taken from the Ensembl genomic database (http://www.ensembl.org/index.html; release 77). The *Ciona savignyi* Fn1 sequence was provided by Tucker, and the cephalochordate tenascin-like sequence was downloaded from GenBank. Species names, abbreviations and accession numbers for the FN1 sequences used in this paper are provided in Additional file 13: Table 1. Multiple sequence alignments (MSAs) were prepared by the progressive iterative alignment method, MUSCLE [81], as implemented in the MEGA6 package of genetic analysis programs [82]. For some analyses, highly gapped unaligned segments were removed from the MSA. Search of the optimal amino acid substitution model was performed in MEGA6 by finding the models with the lowest Bayesian information criterion scores, highest maximum likelihood (ML) value and lowest number of parameters [85]. For the FN protein dataset, we employed the Whelan and Goldman (WAG) substitution matrix [83] with a discrete gamma distribution and 5 rate categories (gamma parameter = 1.69) to model non-uniformity of evolutionary rates among sequences, assuming that a certain fraction of sites are evolutionarily invariable. Phylogenetic trees (Additional file 14: Figure 4) were generated using amino acid MSAs where any site in which the alignment tool introduced a gap was fully excluded from the analysis only in pairwise comparison (pairwise deletion option). Phylogenetic analyses were conducted by ML in MEGA6. Reliability of branching in the trees was assessed by bootstrapping, with 100 replications, respectively. Evolutionary distances between FN proteins in the MUSCLE MSA were ascertained in MEGA6 using the number of amino acid differences per site between sequences (Additional file 16: Figure 3).

#### CRISPR/Cas9 cloning

U6>*sgRNA(F* + *E)* and Mesp>*nls::Cas9::nls* plasmids were a kind gift of Lionel Christiaen [45]. The previously described *Ci_Brac* enhancer [46] was amplified (BraHO1_SpeIF: 5′- GGGACTAGTACCATCGAGTA-3′ and BraHO1_NotIR: 5′-TTTGCGGCCGCAATTGATTC-3′) and inserted into the Mesp>*nls::Cas9::nls* using SpeI and Not1 sites. Putative (N20) + GG *FN* gRNA targets were identified with Jack Lin’s CRISPR/Cas9 gRNA Finder (http://spot.colorado.edu/~slin/cas9.html) and subsequently screened for off-targets and polymorphisms. gRNAs were inserted into the empty U6>*sgRNA(F* + *E)* plasmid by inverse PCR. A single nucleotide mismatch mutation was introduced by site-directed mutagenesis. Primers are listed in Additional file 15: Table 6. To assess mutagenesis of *FN*, we amplified the presumed CRISPR target region using genomic DNA isolated from pooled transgenic embryos (n>100). Targeted Fibronectin genomic DNA was amplified and cloned into a pCRII-TOPO dual-promoter plasmid (Invitrogen) prior to sequencing (Additional file 17: Table 7).

## Additional files

10.1186/s13227-016-0056-4 Exon Lengths and Splice Phases in the CinFN gene. Exon length and splice phases of the CinFN gene were estimated by BLAT nucleotide searches of the C. intestinalis genome (Joint Genome Institute v2.0) with the UCSC Genome Browser. Exons are color-coded according to their splice phases.
10.1186/s13227-016-0056-4 Intron Lengths in the *CinFN* Gene. Intron lengths in the *CinFN* gene were estimated by BLAT nucleotide searches of the *C. intestinalis* genome (Joint Genome Institute v2.0) with the UCSC Genome Browser.
10.1186/s13227-016-0056-4 Domain Structure of the CinFN protein. Domain identification was performed online with the SMART7 tool. Candidate domains were validated by BLAST comparisons against the full chordate protein database and/or human FN1 sequence, and then mapped against the full-length CinFN protein sequence.
10.1186/s13227-016-0056-4 Estimates of Evolutionary Divergence between FN Sequences. Evolutionary divergence between tunicate and vertebrate FN protein sequences was calculated in MEGA6 as the number of amino acid differences per site from between sequences. Pairwise differences are shown below the diagonal, and analytical standard error estimates above the diagonal. The analysis involved 11 amino acid sequences with 4516 positions. All ambiguous positions were removed for each sequence pair.
10.1186/s13227-016-0056-4 pFN2>GFP reporter expression in late stage larvae. Representative pFN2>GFP transgenic larvae illustrating the relative strength of reporter expression in cells at the proximal end of the notochord. (A) High gain and (B) low gain images to display relatively high fluorescence levels in 2 proximal cells.
10.1186/s13227-016-0056-4 Targeted RNAi knockdown of FN generates defects in notochord morphogenesis. (A-D) Representative Bra:FNHP1998 phalloidin stained embryos fixed at approximately 12 HPF. (A) Bra:GFP negative control condition. (B-D) FN knockdown embryos representative of each hairpin phenotype: (B) “Normal” (C) “Mildly Defective” and (D) “Severely Defective.” Scale bar: 20 μm. (E) Bar graphs comparing the percentage of each phenotype in Bra:GFP vs. Bra:ScFNHP1998 vs Bra:FNHP1998 samples. N>300 per condition spanning at least four trials. Arrow bars: standard error, (P<0.0001). (Statistical analysis: A chi-squared test was first conducted to determine whether or not overall distribution of phenotypic categories (normal, mild, severe) was significantly different between Brac>GFP, Brac>scFNHP1998, and Brac>FNHP1998 data (X2 = 861.65, df = 6, p < 2.2e-16). Then, to determine significance for comparisons between control and experimental samples, we conducted two-tailed, two-sample proportion test. Severely defective Brac>GFP vs Brac>FNHP1998 (Z=18.0549, df=1, p < 2.2e-16). Mildly defective Brac>scFNHP1998 vs Brac>FNHP1998 (Z=-10.1005, df=1, p< 2.2e-16). **Movies 1–6.** Time lapse confocal projections of representative U6>*FngRNA6*, Brac>*nls::Cas9::nls*, Brac>GFP transgenic embryos. Membranes stained by incubation with FM4-64 (50μl of 100μg/1 ml H20 stock solution added to 1 ml FSW in a cover-slip bottom imaging dish (MatTek). Samples imaged at 10-minute intervals (Movies 1-4) or 1-minute intervals (Movies 5-6).
10.1186/s13227-016-0056-4: Representative early stage embryo used to evaluate invagination.
10.1186/s13227-016-0056-4 Orthogonal section through embryo in Movie 1.
10.1186/s13227-016-0056-4 Optical section through embryo with bi-lateral incorporation of Brac-GFP, as analyzed in Figure 4C-F’.
10.1186/s13227-016-0056-4 Optical section through additional embryo with bi-lateral incorporation of Brac-GFP, note active jostling of intercalation defective cells.
10.1186/s13227-016-0056-4 Optical section through embryo with unilateral incorporation of Brac-GFP, as analyzed in Figure 4G-J’. Movie 4. Red and green channels. Movie 5. Green channel alone.
10.1186/s13227-016-0056-4 Optical section through embryo with unilateral incorporation of Brac-GFP, as analyzed in Figure 4G-J’. Movie 4. Red and green channels. Movie 5. Green channel alone.
10.1186/s13227-016-0056-4 Gene names, abbreviations, species, and genomic locations of the Fibronectin sequences used in this article.
10.1186/s13227-016-0056-4 Phylogenetic relationships within the FN protein family. Evolutionary relatedness was inferred by maximum likelihood of protein sequences with a Whelan and Goldman + Frequencies substitution matrix and a gamma distribution to model evolutionary rate differences among sites (α = 1.69) using a MUSCLE alignment. The amphioxus (Branchiostoma) FN tenascin sequence was used as the outgroup. The tree with the highest log likelihood (-52556.4682) is shown. Reliability of branching was assessed by bootstrapping (100 pseudo-replicates) with the percentage of trees in which the associated taxa clustered together shown next to the nodes. Branch length is proportional to the number of expected substitutions per amino acid position (shown below the branches). The scale bar represents 0.5 expected substitution per site.
10.1186/s13227-016-0056-4 List of primers used for CRISPR/CAS9 cloning.
10.1186/s13227-016-0056-4 FN protein alignment. Alignment of the vertebrate andtunicate FN proteins mouse and human MAGP-2 promoters. Sequences were aligned using MUSCLE in MEGA6. Amino acids are color-coded according to physicochemical properties.
10.1186/s13227-016-0056-4 List of primers used for qPCR and reporter constructs.

## Abbreviations

FN: fibronectin
ECM: extracellular matrix
SL: splice leader
HPF: hours post-fertilization
GRN: gene regulatory network
PCP: planar cell polarity
