# Supplementary figures and images for: Fibronectin contributes to notochord intercalation in the invertebrate chordate, Ciona intestinalis
Source: EvoDevo. 2016 Aug 31;7(1):21. doi: 10.1186/s13227-016-0056-4 (PMC5006582; doi:10.1186/s13227-016-0056-4)

**A**

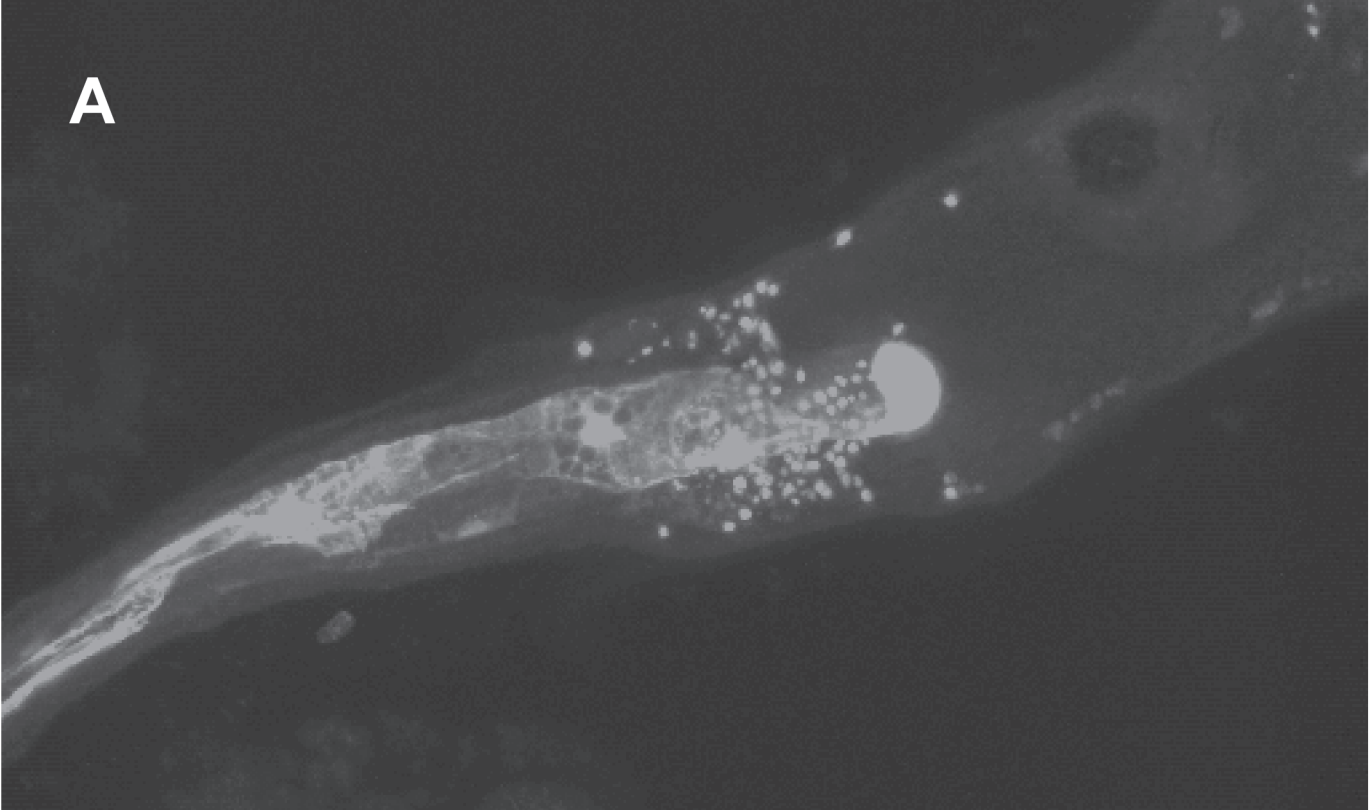

**B**

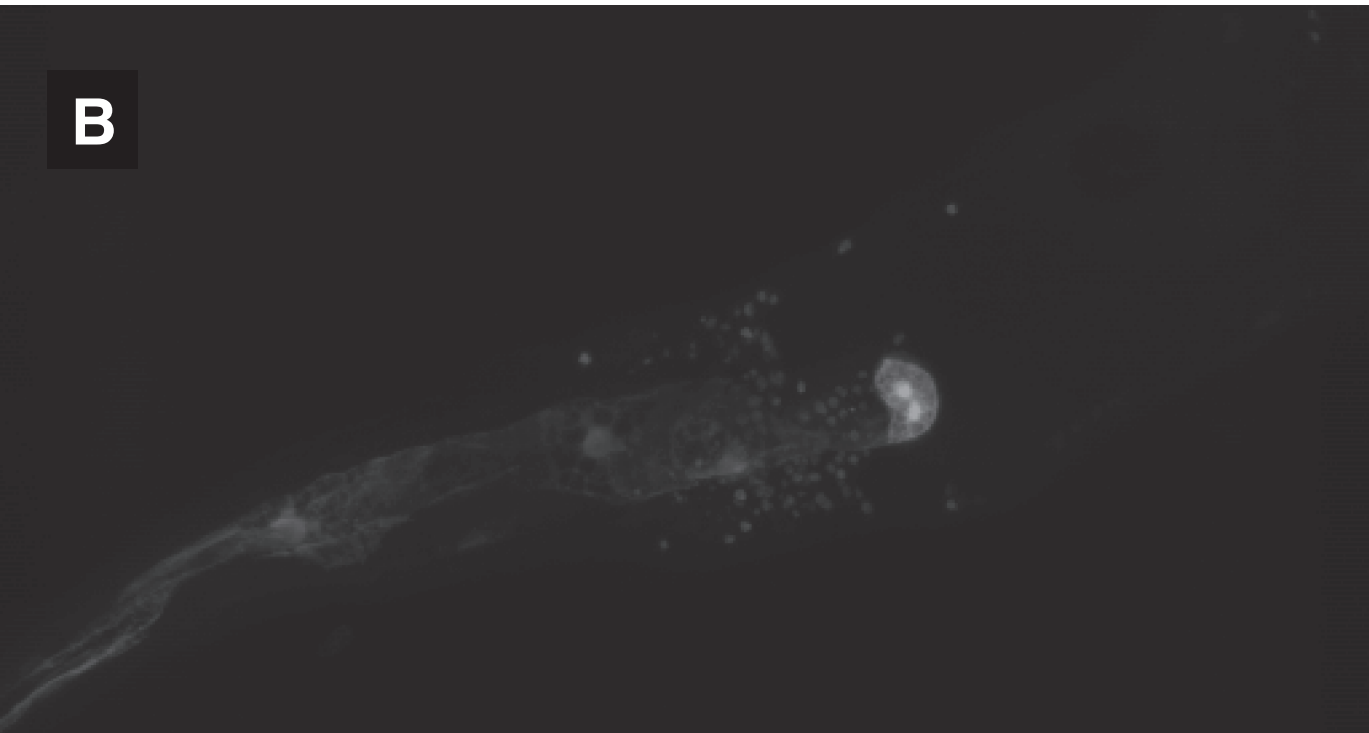

Supplement: Supplementary file 5 — 10.1186/s13227-016-0056-4 pFN2>GFP reporter expression in late stage larvae. Representative pFN2>GFP transgenic larvae illustrating the relative strength of reporter expression in cells at the proximal end of the notochord. (A) High gain and (B) low gain images to display relatively high fluorescence levels in 2 proximal cells. [file 13227_2016_56_MOESM5_ESM.pdf]

**A**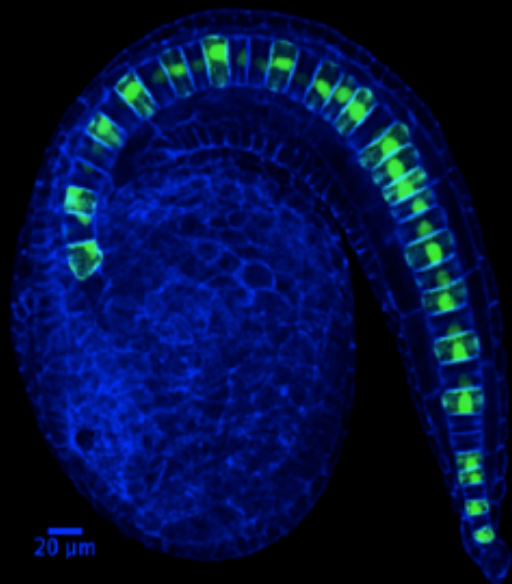**B**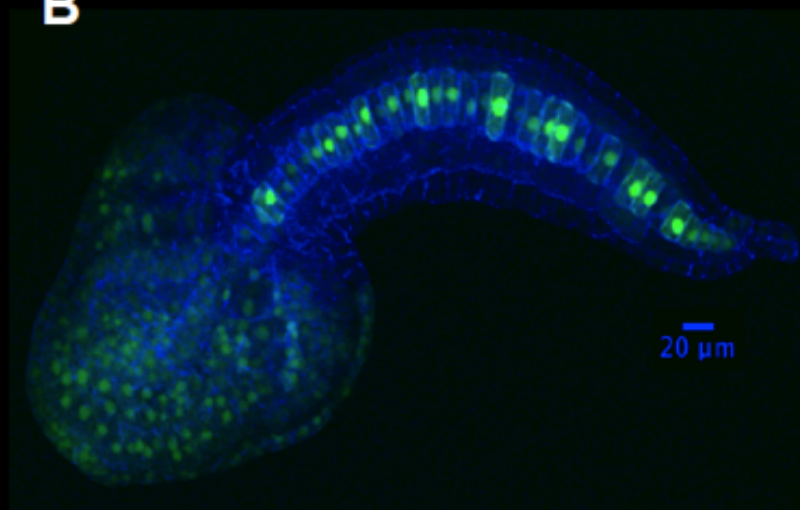**C**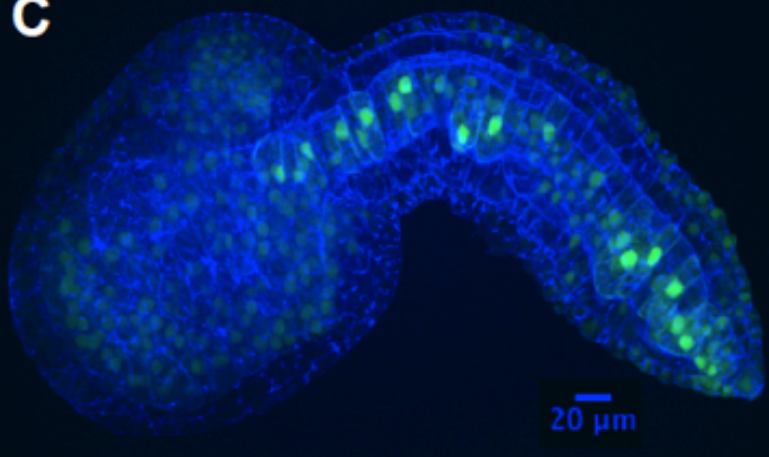**D**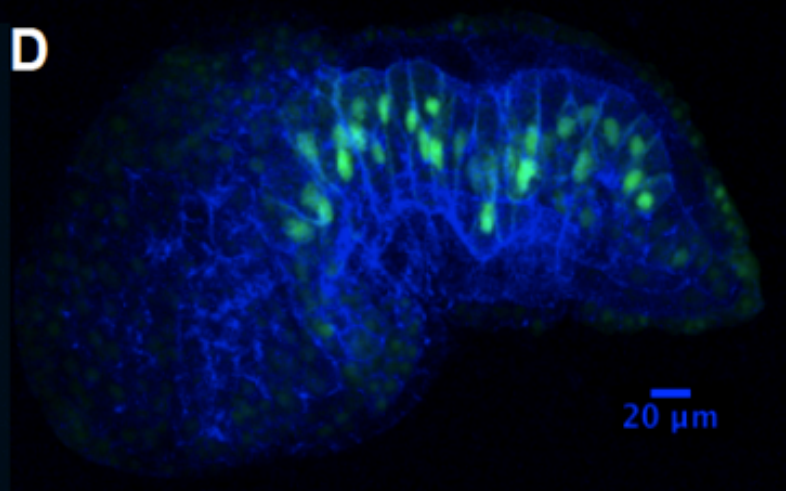**E**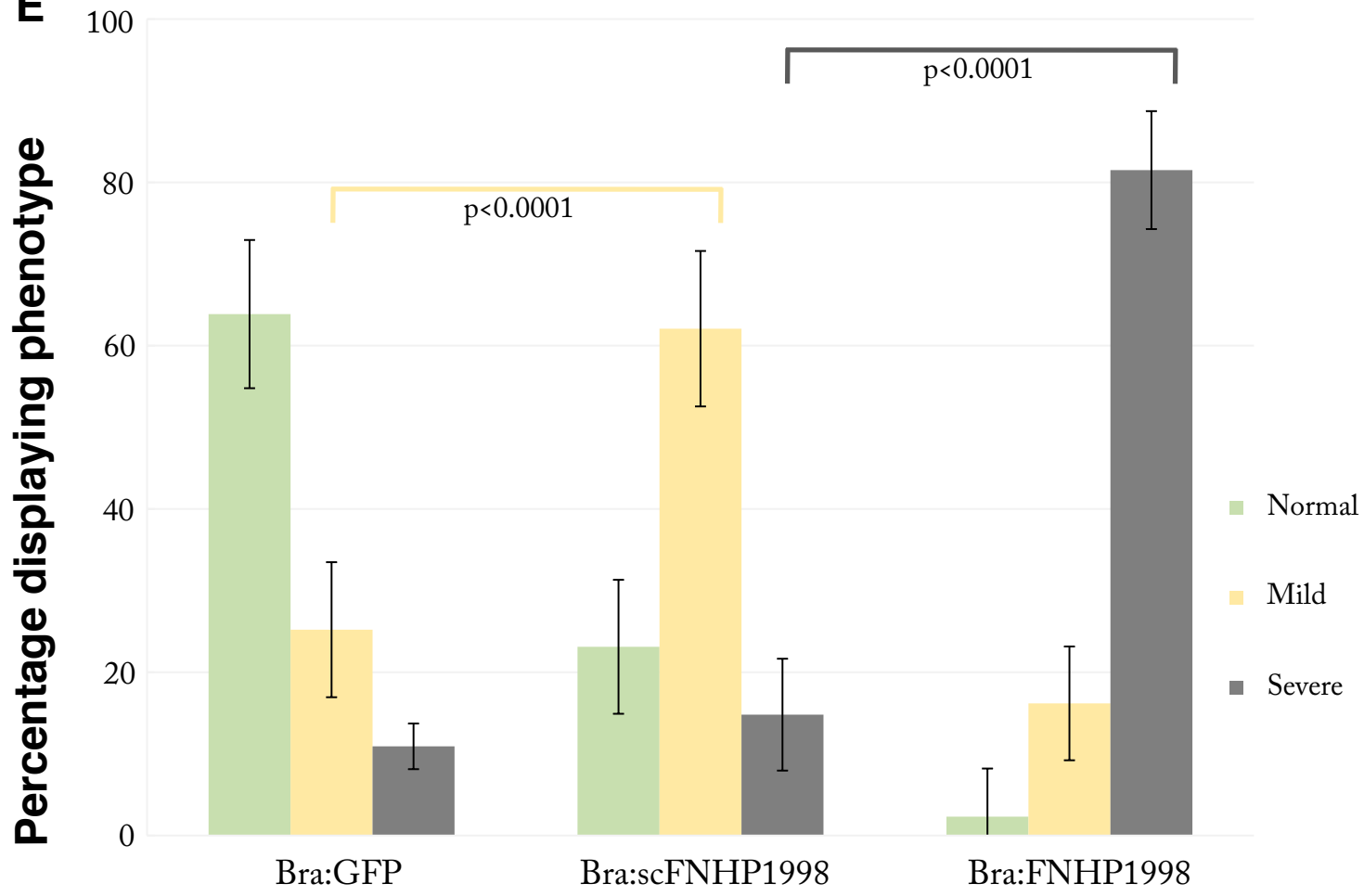

Supplement: Supplementary file 6 — 10.1186/s13227-016-0056-4 Targeted RNAi knockdown of FN generates defects in notochord morphogenesis. (A-D) Representative Bra:FNHP1998 phalloidin stained embryos fixed at approximately 12 HPF. (A) Bra:GFP negative control condition. (B-D) FN knockdown embryos representative of each hairpin phenotype: (B) “Normal” (C) “Mildly Defective” and (D) “Severely Defective.” Scale bar: 20 μm. (E) Bar graphs comparing the percentage of each phenotype in Bra:GFP vs. Bra:ScFNHP1998 vs Bra:FNHP1998 samples. N>300 per condition spanning at least four trials. Arrow bars: standard error, (P<0.0001). (Statistical analysis: A chi-squared test was first conducted to determine whether or not overall distribution of phenotypic categories (normal, mild, severe) was significantly different between Brac>GFP, Brac>scFNHP1998, and Brac>FNHP1998 data (X2 = 861.65, df = 6, p < 2.2e-16). Then, to determine significance for comparisons between control and experimental samples, we conducted two-tailed, two-sample proportion test. Severely defective Brac>GFP vs Brac>FNHP1998 (Z=18.0549, df=1, p < 2.2e-16). Mildly defective Brac>scFNHP1998 vs Brac>FNHP1998 (Z=-10.1005, df=1, p< 2.2e-16). Movies 1–6. Time lapse confocal projections of representative U6>FngRNA6, Brac>nls::Cas9::nls, Brac>GFP transgenic embryos. Membranes stained by incubation with FM4-64 (50μl of 100μg/1 ml H20 stock solution added to 1 ml FSW in a cover-slip bottom imaging dish (MatTek). Samples imaged at 10-minute intervals (Movies 1-4) or 1-minute intervals (Movies 5-6). [file 13227_2016_56_MOESM6_ESM.pdf]
