# Supplementary material for: Fibronectin contributes to notochord intercalation in the invertebrate chordate, Ciona intestinalis
Source: EvoDevo. 2016 Aug 31;7(1):21. doi: 10.1186/s13227-016-0056-4 (PMC5006582; doi:10.1186/s13227-016-0056-4)
Supplement: Supplementary file 16 — 10.1186/s13227-016-0056-4 FN protein alignment. Alignment of the vertebrate andtunicate FN proteins mouse and human MAGP-2 promoters. Sequences were aligned using MUSCLE in MEGA6. Amino acids are color-coded according to physicochemical properties. [file 13227_2016_56_MOESM16_ESM.pdf]

```
Alignment: C:\Users\Fernando\Documents\FS Science stuff\Fibronectin paper\Sequence analysis\MEGA analysis for protein\F
Seaview [blocks=10 fontsize=8 A4] on Wed Oct 22 16:59:33 2014

              1
HsaFN1     -----SKSKR QAQQMVQP-- ---QSPVAVS QSKPGCYD-N GKHYQINQQW ERTYLGNALV CTCYGGSRGF NCESKPEAEE TCFDKYTGNT
MonFN1     -----TKKRR QTQQMIQP-- ---PSPVAVS QSKTGCYD-N GKHYQINQQW ERTYLGNALV CTCYGGSRGF NCESKPEAEE TCFDKYTGNT
GalFN1     GQRRGGKNRR QAQTASVP-- ---QA--TPA QGKQTCFD-N GRYYQINQQW ERIYLGNTLV CTCYGGSRGF NCESKPEPEE TCFDKYTGST
AnoFN1     ---PKDKSKR QAQQQQIV-- ---QPALPAA QNKPGCYD-N GQHYQINQQW ERIYLGNTLV CTCYGGSRGF NCESKPEAEE TCFDKYTGVT
XenFN1     -----SKKRR QAQQQQVV-- ---QPHGSPD SQQKGCYD-N GKYYQINQQW ERTYLGNTLV CTCYGGGRGF NCESKPESEE TCFDKYTGVT
DanFN1a    GT---GKHKR QAQEHSVS-- ---------S VSQDGCVM-D GQFYGAGEKW ERIYLGSTLL CTCHGVSK-I ECKSKPDAEE TCYDKVNSRS
DanFN1b    ---SAGKSKR QAQQQQIHLD TVYEEARSLA IHESGCND-N GRVYRMNDEW ERPYMDSTLK CTCEGAS-GV KCRSKPAAEE SCYDKFNARS
PetFN1     ---------- ---------- ---------- ----GCNV-N GKMYSVAERW ERVYLGRMLT CTCFGGDRGW TCEGKPEP-E SCFDQYTGGR
PetFN2     -----ARRGR GAAPLHLR-- ---------- -GER-----A GP-VDVRAQR S--------- ---------- ---------- --WR------
CsaFN      ---------- ---------- ---TPLSHRG YGNVRCKDKD GTFYNVGTNF IRQR--DNYI CNCTVDYE-- NLRTRLSCAS SAYRLFSTSS
CinFN      ---------T PLSYQSVP-- --------RG NENIHCKDKD GTTYNVGYNF VRAR--DNYV CNCTVDY--V NLVTRLSCSS QNYGRFSSRR

             91
HsaFN1     YRVGDTYERP KDSMIWDCTC IGAGRGRISC TIANRCHEGG QSYKIGDTWR RPHETGGYML ECVCLGNGKG EWTCKPIA-- -EKCFDHAAG
MonFN1     YRVGDTYERP KDSMIWDCTC IGAGRGRISC TIANRCHEGG QSYKIGDTWR RPHETGGYML ECVCLGNGKG EWTCKPVA-- -ERCYDNTAG
GalFN1     YRVGETYERP KDSMIWDCTC IGAGRGRISC TIANRCHEGG KSYKIGDTWR RPHETGGYLL ECVCLGNGKG EWTCKPLA-- -ERCYDNTAG
AnoFN1     YRVGETYERP KDSMIWDCTC IGAGRGRISC TIANRCHEGG QSYKIGDTWR RPHETGGYML ECVCLGNGKG EWTCKPVA-- -ERCYDNSAG
XenFN1     YRVGETYERP KDNMIWDCTC IGAGRGRISC TIANRCHEGG QSYKIGDTWR RPHETGGYML ECVCLGNGKG EWTCKPVA-- -ERCYDNTAG
DanFN1a    YRVGETYERP KDGMIWDCTC IGSARGKISC TIANRCHEGG HSYKIGDTWQ RPHDTGDYML ECVCLGNGKG EWTCKPVA-- -ERCYDNTAG
DanFN1b    YRVGETYERP KDNMIWDCTC IGSGKGKISC TIANRCHEGG NSYRIGDTWT RPHDTGDYML ECVCLGNGKG EWTCKPVA-- -ERCYDDSLG
PetFN1     YRAGETWERP KGGLVWDCTC LGASVSRISC TTASRCHEGG SSYKIGDKWR RVHESGSYMM DCVCLGNGKG EWICNPVSGS QERCYDNTLG
PetFN2     -RPGPDPDHP ---------- -GKSQGPQ-A ARRTRCRLG- ---------- ---------- ---------- ---------- --------RG
CsaFN      NRNNGYTEHA R--------- ---------S ---------- ---------- ---------- ---------- ---------- ----------
CinFN      YRPGYT-ERA R--------- ---------S MQADHCEFNG QLYQLNTRWK MQYN--GMLV DCIC--NATA RLTCYRSF-- -SSYFNQVGQ

            181
HsaFN1     TSYVVGETWE KPYQGWMMVD CTCLGEGSGR ITCTSRNRCN DQDT-RTSYR IGDTWSKKDN RGNLLQCICT GNGRGEWKCE RHTSVQTT-S
MonFN1     TSYVVGETWE KPYQGWMMVD CTCLGEGSGR ITCTSRNRCN DQDT-KTSYR IGDTWSKKDN RGNLLQCICT GNGRGEWKCE RHSSLQTT-A
GalFN1     TSYVVGETWE KPYQGWMMVD CTCLGEGSGR ITCTSRNRCN DQDT-KTSYR IGDTWSKKDN RGNLLQCICT GNGRGEWKCE RHTSLHTT-S
AnoFN1     TSYLVGEIWE KPYQGWMIVD CTCLGEGSGR ITCTSRNRCN DPDT-KTSYR IGDTWSKKDN RGNLLQCICT GNGRGEWKCE RHSSLQTT-G
XenFN1     ISYVVGQTWE KPYQGWMMVD CTCLGEGSGR ITCSSKNRCN DQDT-RTSYR IGNSWSKTDT RGNLLQCICT GNGRGEWKCD RHSSAQAT-G
DanFN1a    TSYMVGETWE KPYQGWMIVD CTCLGEGSGR ITCTSRNRCN DQDT-RTSYR IGDTWSKTDS RGHVLQCLCT GNGRGEWKCE RHASLHTT-S
DanFN1b    SSYVVGQTWQ KPYQGWMIVD CTCLGEGNGR ITCTSRNRCN DQDT-RTSYR IGETWSKIDS SGHTLQCLCT GNGRGEWKCD RHAASHVIPA
PetFN1     SAHLVGDTWE RPYQGWMVVD CTCLGEGQGR IACTSRNRCN DQET-RRSYK VGETWSRADA RGQPLRCLCI GNGNGEWKCD RQTSSVTA-T
PetFN2     VEFVSGQRWT R--------- ----AQGQHR EICS------ ---------- ---------- ------CSAG GEANADA--- ----------
CsaFN      ---------- ---------- ---------- ---------- ---------- ---------- ---------- --EHSRSLA- ----------
CinFN      SGVGVNSVVN QP-------- ---------- --VPQRERSS DQLCLDGTYI LGEDFAVLQP GNFYSICHCT GNSHNPSTC- ----------

            271
HsaFN1     SGSG---PFT DVRAAVYQPQ P-HPQPP--- --PYGHCVTD SGVVYSVGMQ WLKTQ-GNKQ MLCTCLGNGV SCQETAV--- ----------
MonFN1     TGPG---TFT DVQTALYQPQ P-QPQPV--- --PYGHCVTD SGVVYSLGMQ WLKTQ-GNKQ MLCTCLGNGV SCQETAV--- ----------
GalFN1     TGSGSP-SFT NVQTALYQPQ PQQPQPQ--- --PHGHCVTD NGVVYSLGMQ WLKTQ-GSQQ MLCTCLGNGV SCQEI----- ----------
AnoFN1     IGTGSA-TIT DVRTALYQPQ P---QPA--- --PYGHCVTD NGMVYFLGMQ WLKTQ-GSQH MLCTCLGNGV SCQETTV--- ----------
XenFN1     TGSN---PIT NIQTTLFQPD S---ELE--- --PYGHCVTD NGVLYSLGMR WLKAQ-GSKQ MLCTCLGNGV SCEETVE--- ----------
DanFN1a    LGTGSR-VVT NVQPAVYHPQ G-VPEHP--- --VEGSCLTE AGVSYAPGMR WSKAQ-GSKQ MLCTCLGNGV SCEESESQ-- ----------
DanFN1b    IGTGS--AVT HRVTPVMNQV NVLNELI--- --EEGNCKTD SGVSYYNGMS WIRTQ-GTKE MLCTCVGGGI SCEEQDGQ-- ----------
PetFN1     VSRSSRRWFV G---GVRPPP PPLSLSVGTV YRPTVQCEAD GGALFSSGER WVKSQDGGGQ LLCTCLPSGV NCQTIGPP-- ----------
PetFN2     ---------- ---------- ---------- ---------- -------GM- ---------- ---------- ---------- ----------
CsaFN      ---------- ---------- ---------- ---------- ---------- ---------- ---------- ---------- ----------
CinFN      ---------- ---------- ---------- ---------- ---------- ---------- ---------- --------FA VLQPGNFYSI

            361
HsaFN1     ---------- -QTYGGNSNG EPCVLPFTYN GRTFYSCTTE GRQDGHLWCS TTSNYEQDQK YSFCTDHTVL -------VQT RGGNSNGALC
MonFN1     ---------- -QTYGGNSNG EPCVLPFTYN GRTYYSCTTE GRQDGHLWCS TTSNYEQDQK YSFCTDHTVL -------VQT RGGNSNGALC
GalFN1     ---------- ---------- ---------- ---------- ---------- ---------- --------VL -------VQT RGGNSNGALC
AnoFN1     ---------- -QTYGGNSDG EPCALPFTYN GRTFYSCTSE GRNDGTLWCS TTSNFDQDQR YSFCTEQNVL -------IQT RGGNSNGALC
XenFN1     ---------- -ITFGGNANG EPCAIPFTHD GKTYYSCTGE GRQDGKLWCA TTSNYDTDKK YSFCNEQRAL -------VQT RGGNSNGALC
DanFN1a    ---------- -QVYGGTSGG EPCAFPFVFM GKTFYSCTSE GRNDGQLWCS TSSDFEKDYK YSFCTSNNVV -------VTT RGGNSNGALC
DanFN1b    ---------- -QVYGGNSGG QPCVFPFVFS GNTHYSCISE GRSDGQLWCS TTSDYDSDGL YSFCTQKNLL -------VTT RGGNSNGALC
PetFN1     ---------- -KTHGGNSNG APGVSPFTHK GRGHTTCTAK GRDNGKLWGS TTANYPQNKK FSLGSQKKGT T---VTLVTT RGGNSNGALC
PetFN2     ---------- -RTRGGNADG DPCVFPFVFE GMALWGCTAR GRTDGRQWCG TTSNFDRDSR YTYCVDEHAE M---L--VPS QGGNSHGALC
CsaFN      ---------- ---AVLTVSG QECKFPFEES GRLYSTCTFH -RLNRVPYCA TSAIHSQQLE ISNCITSK-- -------VFT ERGNANGARC
CinFN      CHCTGNSHNP STCFVLTVSG TSCQFPFEEN GMLFSTCTSH GLRQ-PPHCA TSPRESSQLT VSNCITTKQA FNQYFGFVFT DGGNANGARC

            451
HsaFN1     HFPFLYNNHN YTDCTSEGRR DNMKWCGTTQ NYDADQKFGF CQTYGGNSNG EPCVLPFTYN GRTFYSCTTE GRQDGHLWCS TTSNYEQDQK
MonFN1     HFPFLYNNRN YTDCTSEGRR DNMKWCGTTL NYDADQKFGF CQTYGGNSNG EPCVLPFTYN GRTYYSCTTE GRQDGHLWCS TTSNYEQDQK
GalFN1     HFPFLYNNRN YTDCTSEGRR DNMKWCGTTE NYDADQKFGF C--------- ---------- ---------- ---------- ----------
AnoFN1     HFPFLYNNRN YTDCTSEGRR DNMKWCGTTH NYDGDQKFGF CQTYGGNSDG EPCALPFTYN GRTFYSCTSE GRNDGTLWCS TTSNFDQDQR
XenFN1     NFPFLYNNRN YTDCTSEGRR DSMKWCGTTA NYDADQKFGF CITFGGNANG EPCAIPFTHD GKTYYSCTGE GRQDGKLWCA TTSNYDTDKK
DanFN1a    QFPFLYNGRN YTDCTADGRR DGMKWCGTTY NFDKEQRFGF CQVYGGTSGG EPCAFPFVFM GKTFYSCTSE GRNDGQLWCS TSSDFEKDYK
DanFN1b    QFPFKYNGRN YTDCTAEGRR DGMKWCGTTA DYDREQKYGF CQVYGGNSGG QPCVFPFVFS GNTHYSCISE GRSDGQLWCS TTSDYDSDGL
PetFN1     HFPFVFGGRE HVACTSEGRA DGMKWCSTTA NFDTDQRYGF CKTHGGNSNG APGVSPFTHK GRGHTTCTAK GRDNGKLWGS TTANYPQNKK
PetFN2     ALPFSFGGRV YSECTAVGRD DGELWCSTTA DFDGDGKYGF CRTRGGNADG DPCVFPFVFE GMALWGCTAR GRTDGRQWCG TTSNFDRDSR
CsaFN      HFPFLFRGVE YYDCITTGRQ AP--WCATTH DYDRDSRYGY C--------- ---------- ---------- ---------- ----------
CinFN      HFPFVVRGVE YHNCITTGQR RG--WCATTS NYERDGRYGF C--------- ---------- ---------- ---------- ----------

            541
HsaFN1     YSFCTDHTV- ---LVQTRGG NSNGALCHFP FLYNNHNYTD CTSEGRRDNM KWCGTTQNYD ADQKFGFCPM AAHEEICTTN EGVMYRIGDQ
MonFN1     YSFCTDHTV- ---LVQTRGG NSNGALCHFP FLYNNRNYTD CTSEGRRDNM KWCGTTLNYD ADQKFGFCPM AAHEEICTTN EGVMYRIGDQ
GalFN1     --------V- ---LVQTRGG NSNGALCHFP FLYNNRNYTD CTSEGRRDNM KWCGTTENYD ADQKFGFCPM AAHEEICTTN DGIMYRVGDQ
AnoFN1     YSFCTEQNV- ---LIQTRGG NSNGALCHFP FLYNNRNYTD CTSEGRRDNM KWCGTTHNYD GDQKFGFCPM AAHEEICTTN EGVMYRVGDQ
XenFN1     YSFCNEQRA- ---LVQTRGG NSNGALCNFP FLYNNRNYTD CTSEGRRDSM KWCGTTANYD ADQKFGFCPM AAHEEICTTN EGVMYRVGDQ
DanFN1a    YSFCTSNNV- ---VVTTRGG NSNGALCQFP FLYNGRNYTD CTADGRRDGM KWCGTTYNFD KEQRFGFCPM AAHEEVCTTN EGVMYRVGDQ
DanFN1b    YSFCTQKNL- ---LVTTRGG NSNGALCQFP FKYNGRNYTD CTAEGRRDGM KWCGTTADYD REQKYGFCPM AAHEEVCTVN D-VMYRVGDE
PetFN1     FSLGSQKKGT TVTLVTTRGG NSNGALCHFP FVFGGREHVA CTSEGRADGM KWCSTTANFD TDQRYGFCPM AAHEDVCTA- DGVQHRVGDQ
PetFN2     YTYCVDEHAE M--LVPSQGG NSHGALCALP FSFGGRVYSE CTAVGRDDGE LWCSTTADFD GDGKYGFCPM NGESARGSTR ECLLLAIGTR
CsaFN      ---------- ---------- ---------- ---------- ---------- ---------- ---------- ---------- ----------
CinFN      ---------- ---------- ---------- ---------- ---------- ---------- ---------- ---------- ----------
```

```
        631
HsaFN1   WDKQHD-MGH MMRCTCVGNG RGEWTCIAYS QLRDQCIVDD ITYNVNDTFH KRHEEGHMLN CTCFGQGRGR WKCDPVDQCQ DSETGTFYQI
MonFN1   WDKQHD-MGH MMRCTCVGNG RGEWTCVAYS QLRDQCIVDG ITYNVNDTFH KRHEEGHMLN CTCFGQGRGR WKCDPIDQCQ DSETRTFYQI
GalFN1   WDKQHD-MGH MMRCTCVGNG RGEWTCIAYS QLRDQCIVDG ITYDVNQTFH KRHDEGHMLN CTCFGQGRGR WKCDPVDQCQ DSETRTFYQI
AnoFN1   WDKQHE-MGH MMKCTCVGNG RGEWTCVAYS QLRDQCIVDG ITYDVNQTFH KRHEEGHMLN CTCFGEGRGR WQCDPVDQCQ DSESQTFYQI
XenFN1   WDKQHD-QGH MMRCTCVGNG RGEWTCVAYS QLKDQCIVDG ITYDVNSSFT KRHEEGHMMN CTCYGQGRGR WKCDAIDQCQ DTETRQFYQI
DanFN1a  WDRRHDVLGH MMRCTCVGNG RGEWSCIAYS QLKDQCIVDN LTYEVNQTFT KRHDEGYTMN CTCFGQGRGR WKCDAIDQCQ EPETRVFYQI
DanFN1b  WDKRHDTLGH MMRCTCQGNG RGEWNCISHT QLKDQCVVNG QTYDVNETFD KRHDQGYMMN CTCFGQGRGR WKCDAIDQCQ EPETKVFYQI
PetFN1   WDRPHE-LGQ AMRCTCIGGG RGDWTCVPYT KILDQCVIDG RTYNVNQTFL KRHDEGHMMN CTCLGQGRGR WKCDAMDQCQ DTDSRQFYQI
PetFN2   FDSQ----GG FRRCTA---- -----CVPPP ---GQCSVDG VLHESGKSFF KRHEEGHLMN CTCHGRERGM WTCDGLDQCQ DSDSRDYHQV
CsaFN    ---------- ---------- ---------- ---------- ---------- ---------- ---------- ---------- ----------
CinFN    ---------- ---------- ---------- ---------- ---------- ---------- ---------- ---------- ----------

        721
HsaFN1   GDSWEKYVHG VRYQCYCYGR GIGEWHCQPL QT-------- ---------- ---------- ---------- ---------- ----------
MonFN1   GDSWEKYVHA VRYQCYCYGR GIGEWHCQPL QT-------- ---------- ---------- ---------- ---------- ----------
GalFN1   GDSWEKYVHG VRYQCYCYGR GIGEWHCQPL QA-------- ---------- ---------- ---------- ---------- ----------
AnoFN1   GDSWEKHVHG VRYQCYCYGR GIGEWHCQPL SA-------- ---------- ---------- ---------- ---------- ----------
XenFN1   GDSWEKHLQG VRYQCYCYGK GIGEWHCQPL ST-------- ---------- ---------- ---------- ---------- ----------
DanFN1a  GESWDKLIQG IHYRCYCYGN GIGELSCEPQ HS-------- ---------- ---------- ---------- ---------- ----------
DanFN1b  GQTWNKVIQG TPYRCSCYGN GIGEMACEPL Q--------- ---------- ---------- ---------- ---------- ----------
PetFN1   GETWVRLVEG SPYMCICLGK GIGEWSCNPQ TN-------- ---------- ---------- ---------- ---------- ----------
PetFN2   GDTWAKVVDG LEFRCHCYGN GIGEWNCKRT TP-------- ---------- ---------- ---------- ---------- ----------
CsaFN    ---------- ---------- ---------- --QKVRSANS VYNRIGESVT LDCQIDGT-S HNPIIEWYKL TNSGRRLIYS YDTSTGRGIL
CinFN    ---------- ---------- ---------- ---GMRSANS VYNRIGESVT LDCRFPATDT QYPIISWYKL TNEGRQLIYT YNAADGTGRL

        811
HsaFN1   ---------- ---------- ---------- ---------- ---------- ---------- ---------- ---------- ----------
MonFN1   ---------- ---------- ---------- ---------- ---------- ---------- ---------- ---------- ----------
GalFN1   ---------- ---------- ---------- ---------- ---------- ---------- ---------- ---------- ----------
AnoFN1   ---------- ---------- ---------- ---------- ---------- ---------- ---------- ---------- ----------
XenFN1   ---------- ---------- ---------- ---------- ---------- ---------- ---------- ---------- ----------
DanFN1a  ---------- ---------- ---------- ---------- ---------- ---------- ---------- ---------- ----------
DanFN1b  ---------- ---------- ---------- ---------- ---------- ---------- ---------- ---------- ----------
PetFN1   ---------- ---------- ---------- ---------- ---------- ---------- ---------- ---------- ----------
PetFN2   ---------- ---------- ---------- ---------- ---------- ---------- ---------- ---------- ----------
CsaFN    GPSAGNLARR LAVVNSTKLI ISDMNREDAG TFECNVANHH SGSSN-QITK VSVRPNPAPT ELQYRYVPSR NEATFSWK-T NTTHDSVRLE
CinFN    APSADAFAGR LSVVNSTKLV ISDMTTADAG TFECNVVNQA SGGENTQVTE VSV-AAPNPT NLHYTYIPYS NRVTFSWTPP ETEFDSLRLE

        901
HsaFN1   ---------- ---------- ---------- ---------- ---------- ---------- ---------- ---------- ----------
MonFN1   ---------- ---------- ---------- ---------- ---------- ---------- ---------- ---------- ----------
GalFN1   ---------- ---------- ---------- ---------- ---------- ---------- ---------- ---------- ----------
AnoFN1   ---------- ---------- ---------- ---------- ---------- ---------- ---------- ---------- ----------
XenFN1   ---------- ---------- ---------- ---------- ---------- ---------- ---------- ---------- ----------
DanFN1a  ---------- ---------- ---------- ---------- ---------- ---------- ---------- ---------- ----------
DanFN1b  ---------- ---------- ---------- ---------- ---------- ---------- ---------- ---------- ----------
PetFN1   ---------- ---------- ---------- ---------- ---------- ---------- ---------- ---------- ----------
PetFN2   ---------- ---------- ---------- ---------- ---------- ---------- ---------- ---------- ----------
CsaFN    IQKNGSTRLR KIRLPGRQES YTTQDLRRGV NYEIRVFTVS NGQDSSPAII TYIGPR---- ---------- ---------- ----------
CinFN    LQEHGSNRVR RVRLRRGRTS YTTNELNVGV NYIVRLFTIY NGRDSVPAVI NYIGPRIPRN DISEASIAPE PVVANDEVSI RWSPDAYPNI

        991
HsaFN1   ---------- ---------- ---------- ---------- ---------- ---------- ---------- ---------- ----------
MonFN1   ---------- ---------- ---------- ---------- ---------- ---------- ---------- ---------- ----------
GalFN1   ---------- ---------- ---------- ---------- ---------- ---------- ---------- ---------- ----------
AnoFN1   ---------- ---------- ---------- ---------- ---------- ---------- ---------- ---------- ----------
XenFN1   ---------- ---------- ---------- ---------- ---------- ---------- ---------- ---------- ----------
DanFN1a  ---------- ---------- ---------- ---------- ---------- ---------- ---------- ---------- ----------
DanFN1b  ---------- ---------- ---------- ---------- ---------- ---------- ---------- ---------- ----------
PetFN1   ---------- ---------- ---------- ---------- ---------- ---------- ---------- ---------- ----------
PetFN2   ---------- ---------- ---------- ---------- ---------- ---------- ---------- ---------- ----------
CsaFN    ----WLVQYE DVESGEIESF ELPTDATSHS TTNLTPGRRY RVTLYALLGN NTVQ------ ---------- ---------- ----------
CinFN    PATEWLVKYE DVETGNETSV TLPLDVTSHT VDNLLPGRRY RVTLYALVDQ NAIEISSNEV VTEYRPVEIE SPANVAQPGP YTATIRWSPP

       1081
HsaFN1   ---------- ---------- ---------- ---------- ---------- ---------- ---------- ---------- ----------
MonFN1   ---------- ---------- ---------- ---------- ---------- ---------- ---------- ---------- ----------
GalFN1   ---------- ---------- ---------- ---------- ---------- ---------- ---------- ---------- ----------
AnoFN1   ---------- ---------- ---------- ---------- ---------- ---------- ---------- ---------- ----------
XenFN1   ---------- ---------- ---------- ---------- ---------- ---------- ---------- ---------- ----------
DanFN1a  ---------- ---------- ---------- ---------- ---------- ---------- ---------- ---------- ----------
DanFN1b  ---------- ---------- ---------- ---------- ---------- ---------- ---------- ---------- ----------
PetFN1   ---------- ---------- ---------- ---------- ---------- ---------- ---------- ---------- ----------
PetFN2   ---------- ---------- ---------- ---------- ---------- ---------- ---------- ---------- ----------
CsaFN    ---------- ---------- ---------- ---------- ---------- ---------- ---------- ---------- ----------
CinFN    SQDVDQYEVT YRDQNKVNSP WFTQVVTNPY VTLQHLVPGT TYDVRITPIS NHRRGISSHV TLTTDDDHNE GVVLPPSNLQ LTHPTRSSFL

       1171
HsaFN1   ---------- ---------- ---------- ---------- ---------- ---------- ---------- ---------- ----------
MonFN1   ---------- ---------- ---------- ---------- ---------- ---------- ---------- ---------- ----------
GalFN1   ---------- ---------- ---------- ---------- ---------- ---------- ---------- ---------- ----------
AnoFN1   ---------- ---------- ---------- ---------- ---------- ---------- ---------- ---------- ----------
XenFN1   ---------- ---------- ---------- ---------- ---------- ---------- ---------- ---------- ----------
DanFN1a  ---------- ---------- ---------- ---------- ---------- ---------- ---------- ---------- ----------
DanFN1b  ---------- ---------- ---------- ---------- ---------- ---------- ---------- ---------- ----------
PetFN1   ---------- ---------- ---------- ---------- ---------- ---------- ---------- ---------- ----------
PetFN2   ---------- ---------- ---------- ---------- ---------- ---------- ---------- ---------- ----------
CsaFN    ---------- ---------- ---------- ---------- ---------- ---------- ---------- ---------- ----------
CinFN    ATWNSPKPLS STPYRYRIRY EPYPRKATEN PIIRTTSQTR FKVIGLEGGK TYVFYVSTLS GDEESEIVSG IISTINEVCL VNGIWHGNEE
```

```
        1261
HsaFN1  ---------- ---------- ---------- ---------- ---------- ---------- ---------- ---------- ----------
MonFN1  ---------- ---------- ---------- ---------- ---------- ---------- ---------- ---------- ----------
GalFN1  ---------- ---------- ---------- ---------- ---------- ---------- ---------- ---------- ----------
AnoFN1  ---------- ---------- ---------- ---------- ---------- ---------- ---------- ---------- ----------
XenFN1  ---------- ---------- ---------- ---------- ---------- ---------- ---------- ---------- ----------
DanFN1a ---------- ---------- ---------- ---------- ---------- ---------- ---------- ---------- ----------
DanFN1b ---------- ---------- ---------- ---------- ---------- ---------- ---------- ---------- ----------
PetFN1  ---------- ---------- ---------- ---------- ---------- ---------- ---------- ---------- ----------
PetFN2  ---------- ---------- ---------- ---------- ---------- ---------- ---------- ---------- ----------
CsaFN   ---------- ---------- ---------- ---------- ------ESG- ---------- ---------- ---------- ----------
CinFN   LFFKRNRNNY LQNCTCTRSR SGGQWQCDPI LCREDESNQR SWTHVDETGS FMQTCACISG SLKCTYAAPL PSKKVDYYEN DEALLKCPYH

        1351
HsaFN1  ---------- ---------- ---------- ---------- ---------- ---------- ---------- ---------- ----------
MonFN1  ---------- ---------- ---------- ---------- ---------- ---------- ---------- ---------- ----------
GalFN1  ---------- ---------- ---------- ---------- ---------- ---------- ---------- ---------- ----------
AnoFN1  ---------- ---------- ---------- ---------- ---------- ---------- ---------- ---------- ----------
XenFN1  ---------- ---------- ---------- ---------- ---------- ---------- ---------- ---------- ----------
DanFN1a ---------- ---------- ---------- ---------- ---------- ---------- ---------- ---------- ----------
DanFN1b ---------- ---------- ---------- ---------- ---------- ---------- ---------- ---------- ----------
PetFN1  ---------- ---------- ---------- ---------- ---------- ---------- ---------- ---------- ----------
PetFN2  ---------- ---------- ---------- ---------- ---------- ---------- ---------- ---------- ----------
CsaFN   ---------- ---------- ---------- ------AGDL SSRLSLRNHK DLVIDNLQPA DAGVYECHLM YDDVEPTIYY SNISMIR---
CinFN   ATSRTPTIRW YKVVEGERLP IYTYSSSSQQ EYEERYAGDL SGRLGLQDRK DLIIRDLQPR DSGLFECHVE YAGVQPATYY SNVTVISTTS

        1441
HsaFN1  ---------- ---------- ---------- ---------- ---------- ---------- ---------- ---------- ----------
MonFN1  ---------- ---------- ---------- ---------- ---------- ---------- ---------- ---------- ----------
GalFN1  ---------- ---------- ---------- ---------- ---------- ---------- ---------- ---------- ----------
AnoFN1  ---------- ---------- ---------- ---------- ---------- ---------- ---------- ---------- ----------
XenFN1  ---------- ---------- ---------- ---------- ---------- ---------- ---------- ---------- ----------
DanFN1a ---------- ---------- ---------- ---------- ---------- ---------- ---------- ---------- ----------
DanFN1b ---------- ---------- ---------- ---------- ---------- ---------- ---------- ---------- ----------
PetFN1  ---------- ---------- ---------- ---------- ---------- ---------- ---------- ---------- ----------
PetFN2  ---------- ---------- ---------- ---------- ---------- ---------- ---------- ---------- ----------
CsaFN   ---------- ---------- ---------- ---------- ---------- ---------- ---------- ---------- ----------
CinFN   HIPSNSNSNQ PQVTTNTISL NWDDVEDVSH YLVEYQDVTH NEDSRIVSVA QPEVFLDRLH PSTMYRITVI PVVDGIRGQP IKTIIVNTDG

        1531
HsaFN1  ---------- ---------- ---------- ---------- ---------- ---------- ---------- ---------- ----------
MonFN1  ---------- ---------- ---------- ---------- ---------- ---------- ---------- ---------- ----------
GalFN1  ---------- ---------- ---------- ---------- ---------- ---------- ---------- ---------- ----------
AnoFN1  ---------- ---------- ---------- ---------- ---------- ---------- ---------- ---------- ----------
XenFN1  ---------- ---------- ---------- ---------- ---------- ---------- ---------- ---------- ----------
DanFN1a ---------- ---------- ---------- ---------- ---------- ---------- ---------- ---------- ----------
DanFN1b ---------- ---------- ---------- ---------- ---------- ---------- ---------- ---------- ----------
PetFN1  ---------- ---------- ---------- ---------- ---------- ---------- ---------- ---------- ----------
PetFN2  ---------- ---------- ---------- ---------- ---------- ---------- ---------- ---------- ----------
CsaFN   ---------- ---------- --PPSNLRFD ARGRTSVRIL WNAPYRI--N GEVYSYISRV EYLLSDSSEQ PMVVETPNRY LLIQNLTPGS
CinFN   VSVPQSAPQT TTPTAAPISN IAPPRNLRFD GKGRTSVRAL WNAPYATPDS GEQY----RI EYNLRDSPNE PEIIRTSNLY LQVQNLNPGS

        1621
HsaFN1  ---------- ---------- ---------- ---------- ---------- ---------- ---------- ---------- ----------
MonFN1  ---------- ---------- ---------- ---------- ---------- ---------- ---------- ---------- ----------
GalFN1  ---------- ---------- ---------- ---------- ---------- ---------- ---------- ---------- ----------
AnoFN1  ---------- ---------- ---------- ---------- ---------- ---------- ---------- ---------- ----------
XenFN1  ---------- ---------- ---------- ---------- ---------- ---------- ---------- ---------- ----------
DanFN1a ---------- ---------- ---------- ---------- ---------- ---------- ---------- ---------- ----------
DanFN1b ---------- ---------- ---------- ---------- ---------- ---------- ---------- ---------- ----------
PetFN1  ---------- ---------- ---------- ---------- ---------- ---------- ---------- ---------- ----------
PetFN2  ---------- ---------- ---------- ---------- ---------- ---------- ---------- ---------- ----------
CsaFN   TYQFHVYAEL NGARSIPASA TVTTKRIGNI LVQASNDRRF HRVGESARLS CRYDQTTPGD HNIKWMRNDQ DGGFYLIALY NTSTSQLNEL
CinFN   TYQFRVFTEL YGGSSEPAVG LVTTRPIGKI LVQASTDRRS HVKGETAQIY CRYDQHTPGE HEVLWLRNYK NGSFYPLAVY NTSTSRLMES

        1711
HsaFN1  ---------- ---------- ---------- ---------- ---------- -YPSSSGPVE VFITETPSQP NSHP-IQW-N APQPSHISKY
MonFN1  ---------- ---------- ---------- ---------- ---------- -YPGTSGPVQ VIITESPNQP NSHP-IQW-N APEPSHISKY
GalFN1  ---------- ---------- ---------- ---------- ---------- -YAGATGPVQ VIITESTNQP NSHP-IQW-N APKTSHISKY
AnoFN1  ---------- ---------- ---------- ---------- ---------- -YQGSTGPVQ VIITDVPIHP DSHP-VQW-V APESSHIASY
XenFN1  ---------- ---------- ---------- ---------- ---------- -SPAGTGPVQ VIITESSNFP NSHP-IQW-N APQPSHIKNY
DanFN1a ---------- ---------- ---------- ---------- ---------- -ISGGHRPVQ VIISEAGNQP NSHP-IQW-N APASAHITQY
DanFN1b ---------- ---------- ---------- ---------- ---------- -----TAPVR VIITEAGNQP NSHP-IQW-N APPSAHITQY
PetFN1  ---------- ---------- ---------- ---------- ---------- -LNEVKTPIQ VFISDASNQA SSHPRIEWAN TQQLPHVTGY
PetFN2  ---------- ---------- ---------- ---------- ---------- -PEASVQPVQ VMISESPTNR HSHP-VRW-I NPSSSAASGY
CsaFN   ND--EYRGRI LSPQSSQLVI NNLRPSDSAN YKCIV----- ---------- ---------- VINNLRPSDS ANYKCIVKFA TGGGSGTTSL
CinFN   ENVGDYSGRL ISNEPTRLLI QNLRSTDDGT YKCIVKFLTG GGSGSTRITV V--GPPNKVY FTSANRSSEV GNYL-LSW-H VDDVPRATKF

        1801
HsaFN1  ILRWRP--KN SVGRWK---- ---------- ---------- ---------- ---------- --EATI-PGH LN-------- ----------
MonFN1  ILRWKP--KN SPGRWK---- ---------- ---------- ---------- ---------- --EATI-PGH LN-------- ----------
GalFN1  ILRWRP--KI SGRHWK---- ---------- ---------- ---------- ---------- --EATI-PGH LN-------- ----------
AnoFN1  ILRWKP--KN SRIKWK---- ---------- ---------- ---------- ---------- --EATI-PGY QN-------- ----------
XenFN1  ILRWKP--KL KTGPWK---- ---------- ---------- ---------- ---------- --QATI-PGH LN-------- ----------
DanFN1a ILKWRP--KN THIQWM---- ---------- ---------- ---------- ---------- --EVTI-PGH VN-------- ----------
DanFN1b ILKWRV--KN TRTPWK---- ---------- ---------- ---------- ---------- --EVTI-PGH IN-------- ----------
PetFN1  LLRWRQ--KN SRLSWR---- ---------- ---------- ---------- ---------- --EVSV-PAR LN-------- ----------
PetFN2  NLKWRA--KD SEARWR---- ---------- ---------- ---------- ---------- --EVDL-GVK ED-------- ----------
CsaFN   TVVGPPDQRG SQRSYK---- ---------- ---------- ---------- ---------- --HALAPPTR INLIHSSPRQ VSFNVVGPQE
CinFN   YLGVRK--RG SRRTYNFQMA GNARSFLFRN LKEGKTYEGN VISNNRYGNS TSSDFYFSTP STNAPNPPTA VNLIDLSPRR ISFNVDGPPE
```

```
          1891
HsaFN1    ---------- ---------- ---------S YTIKGLKPGV VYEGQLISIQ QYGHQEVTRF DFTTTSTSTP VTSNTV---- --TGETTPFS
MonFN1    ---------- ---------- ---------S YTIKGLRPGV IYEGQLISVQ HYGHKEVTRF DFTTTSTGPP VSTNMV---- --TGETTPPP
GalFN1    ---------- ---------- ---------S YTISGLKPGV VYEGQLISVQ QHGPKEVTRF DFTTTSTTAV TSNTVS---- --G-ETTLLP
AnoFN1    ---------- ---------- ---------S YTISGLRPGI LYEGQLISVQ HYGKREVTRF EFTTSSTTSI STTRVT---- --G-ETTALP
XenFN1    ---------- ---------- ---------S YTISGLKPGI LYEGQLISIL QYGNREVTTF DFTTTSTIHR SSQTET---- --G-ETTPLP
DanFN1a   ---------- ---------- ---------S YTIAGLKPGV TYEGQLISIL RFGRRETTRF DFSTVHGSLE TSEG------ ----ETTQPP
DanFN1b   ---------- ---------- ---------S YTISGLKAGL TYEGQLISIL RYGPREVTRF DFTTTYGSLA KAEG------ ----ETTQPR
PetFN1    ---------- ---------- ---------S YTITGLKPGL TYEGQLITLL VSGLREVTRF EFTTSLVTHH FGG------- -------TVPP
PetFN2    ---------- ---------- ---------G YVVTGLDPGI TYEGQLFTLR PDGHRDVTRF QFSTSATPRA YPGG------ ----SSPPWP
CsaFN     NVQFYKVSYK PEGASDADLE ILEGNNANAP ITLSLLTPNT PYIINVVATN GNRRSPALIT SITTPEVNRL FRINFVLVLI IDTLEVPDVP
CinFN     NVQLYRIYYK PEGTSDAGMQ VIEGR--DNP FTITGLTPNT PYILRLVAVN DDGTSEPLNT AIITPK---- ---------- ----EPPAKP

          1981
HsaFN1    PLVATSESVT EITASSFVVS WVSASDTVSG FRVEYE-LSE EGD----EPQ YLDLPSTATS VNIPDLLPGR KYIVNVYQIS EDGEQSLILS
MonFN1    PVVTTSESVT EITASSFVVS WVSASDTVSG FRVEYE-LSE EGD----EPQ YLDLPSTATS VNIPDLLPGR KYIVNVYQIS EEGEQNLILS
GalFN1    PMVATSESVT EITASSFVVS WVSASDTVSG FRVEYE-LSE EGD----EPQ YLDLPSTATS VNIPDLLPGR KYIVNVYQIS EEGEQNLILS
AnoFN1    SMVSTSESVT EITANSFVVS WVSASDTISG FRVEYE-LSE DGD----EPK YLDLPSTVTS VNIPNLLPGR NYIVRVYQIT EEGKPDLILS
XenFN1    PLVATSESVT EITASSFLVS WVSASDTVSG FRVEYE-LSE DGD----EKR YLELPNTATS VSIPDLLPGR RYNVNVYQIT EEGEKSLILS
DanFN1a   RMVDTSESVT EITSSSFVIS WVSASDTVSG FRVEYE-LSE EGG-QTGQPM ILDLPHSATS VNISELLPGR KYTVNVYEVT GEGEPNLILT
DanFN1b   QVVDTSESVT EITSNSFVIS WVSASDTVSG FRVEYE-LSE EGA----QKR VLDLPRTTTS VNIQDLLPGR RYNVDVYEVN PEGDTKLILT
PetFN1    VLSGASETMT EITSSSFLVS WVSTSSSVSG FRVEYE-LSE DGAVTHRLGR RSDLPNTANS VSLENLLPGR RYHVKVYEVT DGQDDSLILI
PetFN2    WCGHNTN--- ---------- -CRHDDDFLY LSIPVQGITR DGR------M SHDSLLKEKA DLLP---PPV PCNINALPGQ ECHGTFAVVY
CsaFN     VVLSKLEAAR EVVLN----- -MSQVDDVDF YEVSYK-AIG DGN------P TMSQRSTGDV VRME-LEPDT QYQVNVVAVN NAGQSSPLRM
CinFN     VLLQTTEEAR LATLR----- -ISEGDDVDY YVVTYK-AVR SGS----EIM TLESVATNNI IEVP-LEPDT QYQVNIVAVN EAGQSPKLQT

          2071
HsaFN1    TSQTT----A PDAPPDTTVD QVDDTSIVVR WSRPQAPITG YRIVYSPSVE G---S--STE LNLPETANSV TLSDLQPGVQ YNITIYAVEE
MonFN1    TSQTT----A PDAPPDHAVD RVDDTSIVIR WSRPQAPITG YRIVYSPSVE G---S--STE LNLPETANSV TLSDLQPGMQ YNITIYAVEE
GalFN1    TSQTT----A PDAPPEHSVE SVDDTSIVIS WSRPQAPITG YRIVYTPSVE G---S--STE LNLPDTATSV TLSDLMPGVQ YNISIYAVEE
AnoFN1    TSQTT----A PDAPPDHHVE NVDDTSILVS WSRPEAPITG YRIVYTPSVE G---S--STE LNLPDSATSV TLSDLMPGVQ YNISIFAVEE
XenFN1    TTQTT----A PDAPPDHNVE NVDDTSILIK WTKPQAPVTG YRVVYSPSVE G---S--STE LNLPSTATSV SLTELLPGVE YNITIYAVED
DanFN1a   TSQTT----A PDAPSDHEVE DVADTSIRIS WSKPLAPITG YRVVYTPSEE G---SSGSVE LNLPEHSTSL TLGDLRPGVL YNISIFSVEE
DanFN1b   TTQTT----A PDAPTNHEVS NVQETSIVIR WSKPKAPITG YRVVYTPSVE G---S--STE LILPETQTSV TLGDLRPGLS YNISIYSVED
PetFN1    TTQTT----A PDAPQQYEVT EATDTSLVIR WIAPKAPIRG YRIVFTPVEE GSRPELASTE LTLPSESTSV TLSGLAPGVT YNITLTAVGE
PetFN2    VSMYP----- PEPPEGLKVV DMTESSITIG WDRTDEQLTA YRVVLPD--- T---GRAPKE MMLHPGATSV MLLELVSAEA YTVRLYAVSG
CsaFN     FVRTP----P PATPIKLTVL RNQPNQLTLS RGDADHSVDY YILQYQRDNS D---EVLERR F----TDDEV HLDNLTPGSR YRVTLIAVN-
CinFN     FVETPAEQIP PARPTRFDEI TPTWNSANLT WSGSLFAVQY YVFEYQSD-- E---DIFPLK LNTTGPRQSI FVDGLSPDTK YRVSVKAVG-

          2161
HsaFN1    NQESTPVVIQ QE---TTGTP RSDTVPSPRD LQFVEVTDVK VTIMWT---P PESAVTGYR- -----VDVIP VNLPGEHG-- QR---LPISR
MonFN1    NQESTPVFIQ QE---TTGVH RQDNVPPPTD LQFVEVTDVK VTIMWR---P PESAVTGYR- -----VDVVP VNLPGEHG-- QR---LPVSR
GalFN1    HQESTPVFIQ QE---TTGVP RTDEVPSPKD LQFVEVSDVK VTIMWT---P PQSQVSGYR- -----VEAIP VNRPGQHG-- QR---LPITR
AnoFN1    NQESSPIFIQ QT---TTGVP RESTIPSPKD LQFVEVSDVK ITIMWN---P PDSAVTGYR- -----VEVIP VNRPGEHG-- QK---LPVTR
XenFN1    SLESVPIFIQ QG---TTGTP QTVIVPSPTD LQLVEVTDIK ITIMWN---P PQSEVSGYR- -----VVVKP VSPSDRDV-- QN---LPVSR
DanFN1a   NQESEPVFVQ VT---TAGEP LAEEVPSPTD LQFYEVSDSK ITITWT---S PSSEVSGYR- -------VS VGEVGPDGLI EKEVPYPVTQ
DanFN1b   NLESEPLVLQ AS---TAGEQ QPEEIQAPTD LQFYEVTDVK ITITWT---G PPNEVSGYR- -----VTFAP VSTDGQA--- QRPLQLPVTH
PetFN1    TQDSEPVFIQ QSTLPGTGQH RELILAPPTD LQFVAVTETK IVLMWNRPVA PDDAMMGFR- -----IVVTP LGRAQEA--- FR---VPFTE
PetFN2    DAQSRPSSLQ ASTHGDSRSQ RRTRL----- -PFVSVAFTM NTLFQQ---G KSPVRLKVT- --------FP LRYGPQNG-- YS-----ID-
CsaFN     --DGVPSTLA ETYVSTPNQK VKVSIYLPQP AP-VESSQPV VTMLEK---S PTEVVIRIN- DTETNPDYYI LQYQPESG-- GQ-----VLE
CinFN     --EGGHSPFL VDYFYTLTSP QRTNL----- ---------- -TVLDK---L PGEVTFQIDN LSDDDVEYFI VQYQKEDS-- DE-----VIE

          2251
HsaFN1    NTFAE----V TGLSPGVTYY FKVFAV--SH GRESKPLTAQ QTTK------ ---------L DAPTN-LQFV NETDSTVLVR WTPPRAQI-T
MonFN1    NTFAE----I SGLSPGVTYY FKVFAV--NQ GKESKPLTAE QTTK------ ---------L DAPTN-LRFV NETDTTVLVT WTPPRARI-A
GalFN1    SSFAE----V VDLLPGTTYL FKIFAV--SH GRESKPLTGE QTTK------ ---------L DAPTN-LRFI NTTEHSVLVL WTRPRAII-S
AnoFN1    YSFYE----V TELLPGTTYL FKIYAL--GQ GTESEPLTGE QTTK------ ---------L DGPTN-LRIV NETDTTVLVT WRPPRAQI-T
XenFN1    NTFAE----V VNLHPGRTYT FEVYAV--NR GQESEPLAGD FTTK------ ---------L DAPSD-LQFT DVTDSTVVII WRPPQAKI-G
DanFN1a   NAYAE----I THLQPGTLYR FFIFSL--KS GEESEPLVGE QATK------ ---------P DPPTD-IYFS NITEDSAVIV WLPPTAQI-T
DanFN1b   NAYAE----L MRLQPGTLYR FHIYAV--SG GVESEPLIGE KSTK------ ---------P DAPTD-LRFT DITDDSALVI WSFPRAQV-T
PetFN1    HTFAD----M DGLQPGVTYR FEVYAVYHQG GQESQPLVRE QTTPGAPV-- ---------V DAPSD-MRFV EITETSVAIN WTAPQAAL-D
PetFN2    --FVTLNIYV KQSAAGPVVV MFFSGSTIPR NTSSRLENKH GNRR------ ---------L TAPRE-LRVT DVEETALRAE WLPPPGGPIS
CsaFN     RRFTDDEVHL DNLTPGSRYR VTLIAV--ND GVPSTLAETY VSTPNQKVKM SIYLPQPAPV ESPQPVVTML EKSPTEVVIR INDTETNP-D
CinFN     RTFTEEEIHL DNLTPGSTYR VTLIAV--ID GTRKELTQTE ITTP------ --FEPEPN-L PSTVDNLRVT SLSPTRVKIT AEGPRDNI-A

          2341
HsaFN1    GYRLTVGLTR RGQ------- --PRQYNVGP SVSK-YPLRN LQPASEYTVS LVAIKGNQES PKATGVFTT- ---LQPGSSI PPY--NTEVT
MonFN1    GYRLTVNPTR GNQ------- --PKQYNVGP SATQ-YPLRN LQPGSEYTAS LVAVKGNQQS PKQTGVFTT- ---LQPASSI PHY--NTEVT
GalFN1    GYRLTAGPTR GGQ------- --PRTYNVGP SATK-YLLRN LQPGTEYTVY LVAVKEDLQS ARETGVFTT- ---YQPVGSV PPF--NTEVT
AnoFN1    RYQLSVGPTT GGQ------- --PRQHNVGP SATK-YLLRN LLPGTEYTVT LVAVKGNLQS NPETTIFTT- ---LQPRGSV PHY--NTEVT
XenFN1    RYLLSVGQTR GGQ------- --PSQFHINP SATN-HKLES LSPGTEYTIS LVALKGNQQS ASTTGVFST- ---LEPVGSI PPY--NTEVT
DanFN1a   GYRL-FLTVE GSN------- --PKQLRIPG RLSQ-YTLLN LQPDTEYSAT LHAERGNVLS EGSIATFAT- ---TPPMGNA PYF--STDVT
DanFN1b   GYRL-FISIG SSS------- --PKQLRIPG HKSQ-YKLPN LQPDTEYRVT LHSEQGNTLS EGITDTFRT- ---TQPTGNA PRF--NTEVT
PetFN1    SYHLSYGPTR PGA------- GRPKSLAVSP EARH-HTERG LRPATEYTIT LRSVKNGQRS VPLSAIFTT- ---RPLPGSL PEI--ITEIT
PetFN2    GYRVRCLPAA GGEEDEGAAA GHQEEEELPA GTSS-HRLEG LQPGQHYVIT VTAVRGQEHG PPASLSVDT- ---LE-PFVT PDF--KVDVT
CsaFN     YYILQYQPES GGQ------- --VLERRFTD DE---VHLDN LTPGSRYRVT LIAVNDGVPS TLAETYVSTP NQPPEPVQTA PHRIRVNPES
CinFN     QYNVRYAPAD GTE------- --PETSFYTR GSSPVITLSR LNPATRYRVT VSSVYDGLQS SPVTVEFTT- ---KKVQEIT PTNLRVSNVE

          2431
HsaFN1    ETTIVITWTP APR--IGFKL GVRPSQGG-- ---------- -EAPRE---- VTSDS----- ---------- ---GSIVVSG LTPGVEYVYT
MonFN1    ETTIIITWIP APR--IGFKL GVRPSQGG-- ---------- -EAPRE---- VTSES----- ---------- ---GSIVVSG LTPGVEYVYS
GalFN1    ETSIVITWTP APR--IGFKL GVRPSQGG-- ---------- -EAPRE---- VISDS----- ---------- ---GSIVISG LTPGVEYTYS
AnoFN1    ETSIVITWTP APR--IAFKL GVRPSQGG-- ---------- -EAPRE---- VISES----- ---------- ---GSIVISG LTPGVEYIYS
XenFN1    ETTIVVTWTP VPR--IGFKL DVRPSQGG-- ---------- -EAPRE---- VISES----- ---------- ---GSIVISG LTPGVEYMYS
DanFN1a   DTSIIVSWSP LPK--IGYKL TVRPSQGG-- ---------- -EAPRD---- VTSEL----- ---------- ---GSVLISG LTPGVEYTYS
DanFN1b   DTAIIVTWIP VAK--FSYRM SVKPSQGG-- ---------- -ETPRE---- ETSGS----- ---------- ---GRIYISG LTPGLEYIYS
PetFN1    ETSLTFRWKP TASVSVLFKL FI-PSLSS-- ---------- -MVPRT---- ETSES----- ---------- ---GSILIDG LTPGEQYIVT
PetFN2    ETTITISWAS VPG--LVVRV LLVLSEGG-- ---------- -ADPRE---- LSADS----- ---------- ---GELLLPD LTPSTEYTLT
CsaFN     PYEVILRVDG PSD--HITRY HVTYS----- ---------P IDQPHDQVSY FTTDAD---- ---------- ---NEISLNR LQPSTAYRVA
CinFN     DDEVTLEWNP PVDVSLVRRY IVQYKDGSIP KNPYRTRYIP VSTPYITLEN LTPDTQYFIK LRSIGNNRRP SRVAKLTFRT LSPTTTTTTT
```

```
       2521
HsaFN1    IQVLRDGQER DAPIVNKVVT P--------- ---------- ---------- ---------- ---------- ----LSPPTN LHLEANPDTG
MonFN1    ISVLRDGQER DSPIVKKVVT P--------- ---------- ---------- ---------- ---------- ----LSPPAN LHLEANPDTG
GalFN1    LTVLMDGQER ETPIIRRVTT P--------- ---------- ---------- ---------- ---------- ----LSPPTN LRLEPNPDTG
AnoFN1    ITVLTDGQER DAPIVKTVVT P--------- ---------- ---------- ---------- ---------- ----LSPPTN LHLESNPDTG
XenFN1    ISVLKDGVER DIPITKTVVT P--------- ---------- ---------- ---------- ---------- ----VAPPTN LRLQPSRDSA
DanFN1a   VQPVISGQEH GNPITRRVVT P--------- ---------- ---------- ---------- ---------- ----LSPPTD LNLESNPNTG
DanFN1b   LQPLFSGRKH GSPITNKVVT S--------- ---------- ---------- ---------- ---------- ----LSPPTD LNVEPNSVNG
PetFN1    VSILWDGRQI ARPATKTITT L--------- ---------- ---------- ---------- ---------- ----LMPPQD VQVDFNSPSG
PetFN2    IIILLHGVQR GPPIVKLVTT S--------- ---------- ---------- ---------- ---------- ----VPPPRG IEVEPRGDTG
CsaFN     VSSVYNSVKS NPRTVTFTTL P------LPT EPTPPPLSST LVVTPPSP-- ---------- ---------- ----IHPPTH LRVDHRGFS-
CinFN     TSTMATTMAS TRRTTTSEPT PPVEPIRLPI EPNPRPIEPT PMPIEPTPMP IEHTTMPMLR TTPVSATQLT ESLLVPTPIR LQVLRRGFT-

       2611
HsaFN1    VLTVSWERST TPDITGYRIT TTPTNGQQGN SLEEVVHADQ SS-CTFDNLS PGLEYNVSVY TVKDDKESVP ISDTIIPEVP QLTDLSFVDI
MonFN1    ILTVSWEKST TPDITKYRIT TTPTSGQEGY TLEEVVNPDQ NS-CTFENLS PGVEYNVSVY TVKGDQESVP ISDTIIPAVP P---------
GalFN1    ILIVSWDRST TPGISGYRVT TAPTNGQQGS TLEEVVGADQ TS-CTFENLN PGVEYNVSVY AVKDDQESIP ISKTITQAVP P---------
AnoFN1    VLTVSWERST SPDITGYRIT TIPTNGQQGY TLEEAVDADQ TT-CIFEHLS PGVEYNVSVY TVKDHQESLP ISETITQEVP QLTDLSFVDI
XenFN1    TLTVYWDSSI SPGITGYRIT TAPTPMQVGN SLEEEVGPTQ TY-CLFENLS PGVEYNVSVY AVKGEEESSP LSQIFLQEIP QLTDLKYDDV
DanFN1a   KLTVQWNDAN IPDITGYRVT CTPTKGQQGN SLEEFVKAGQ NS-CTLENLS PGVEYNVSVF TVKDDMESVP VFTTVTPDVP KITDLSFINV
DanFN1b   ELNVRWRGTK SPDITGYRVT GTPINGQRGV SLEESVRGDE TS-CILENLS PGVDYNISVY TVKNHLESEP ISTSVTQDVP KVGDLSFVDF
PetFN1    VLTISWQRVA VPELTGYRVR GVPLRGQRGN SLDELLRPDQ TS-FVLDGLT PGVEYNITVA TVKGVLESKP FSTTLTTVTP RPSDLSFTDV
PetFN2    ELRISWIAPE MPEVGGKRSA SWNKQHTYGG ILLKLCTATP SSLCMNRQLR PGTEYEVAVH TIVGEHESVA LMGTQRTAPD PPRELQIVAV
CsaFN     TADLAWRSPA S-DIDGYYIE YDSTFTQHGE PKVQNLDASS TS-TTLTKLI PGTEYQAQVF SKRGTQQSLS STVTFMTQLD SPSNVH-VRP
CinFN     DAEIAWTSPG S-NIDGFLVE YDTTFTRHGE PQTQQLRENV RR-TTLEDLI PGTEYQVKLF SKRGSLQSRP SLVTFMTKLD APSNVH-VSP

       2701
HsaFN1    TDSSIGLRWT PLNSSTI-IG YR-------- ---------- ---------- ---------- ---------- ---------- ----------
MonFN1    ---------- ---------- ---------- ---------- ---------- ---------- ---------- ---------- ----------
GalFN1    ---------- ---------- ---------- ---------- ---------- ---------- ---------- ---------- ----------
AnoFN1    TDTSIALRWT PLNASTI-IG YR-------- ---------- ---------- ---------- ---------- ---------- ----------
XenFN1    TDTSIDLRWT PLNSSTI-IG YR-------- ---------- ---------- ---------- ---------- ---------- ----------
DanFN1a   TDSTIGLSWS PLNSTAV-TG YR-------- ---------- ---------- ---------- ---------- ---------- ----------
DanFN1b   TDTTIGIRWT PLKYQAV-TG YH-------- ---------- ---------- ---------- ---------- ---------- ----------
PetFN1    SDTSLVLRWT PLSVPGI-TG YR-------- ---------- ---------- ---------- ---------- ---------- ----------
PetFN2    SDSSLNLQWL PPAEAAV-SG YRVRCAT--- ---------- ---------- ---------- ---------- ---------- ----------
CsaFN     NDDELIILWQ NPEGPRTGTR FRYRKAS--- ---------- ---------- ---------- ---------- ---------- ----------
CinFN     QSDRLIISWV NPDGPRVGTH FKYRKPNGPW RQVRLAPDVT SYAIPIRSIH DTYEVSITAF GRTIESLPRT LTVHPRLTEP DDRTSKSPVD

       2791
HsaFN1    ---------- ---------- ITVVAAGEGI PIFEDFVDSS VGYYTVTGLE PGIDYDISVI TLINGGE--- ----SAPTTL TQQTA--VPP
MonFN1    ---------- ---------- ---------- ---------- ---------- ---------- ---------- ---------- ----------
GalFN1    ---------- ---------- ---------- ---------- ---------- ---------- ---------- ---------- ----------
AnoFN1    ---------- ---------- ITVVAAGESV PIFEDFVDSS VGYYTITGLE PGIDYEISVI TLINGGE--- ----SAPTTL TQQTA--VPP
XenFN1    ---------- ---------- ITVVAAGESV PIYEEFVGPT DGYYKVSGLE PGIDYEISVI TLISGGE--- ----SAPTTI VQHTA--VPP
DanFN1a   ---------- ---------- ITVLAAGDSV PIFVEFVEPT TGFYTVHGLE PGIDYDITVT TVTENGE--- ----SEPITI TQQTA--VPA
DanFN1b   ---------- ---------- ITVVTSGQNF PILEDVVNSS VNYYTIRGLE PGINYDISVS TITDEAE--- ----SVPSVI TQQTQTAVPA
PetFN1    ---------- ---------- VTVSLPGDSS PLLEDTVGPR TGVYAVEGLR PGVVYDIGVS TLSEDGE--- ----SEPARR SQTTA--VPP
PetFN2    ---------- -------EED PSVMFAASNG REDPRYVSSD SGLMLLPDLS PHAEYTLTII ILLHGTQ--- ----RGPPIV KHVTTAELAD
CsaFN     ---------- ---------- FHVTLQANGT EPRTLDLQPS VTFYQLENLK PTTHYTVLVD NGDQTEQIDS AEFVTKADRV VDFHADQINS
CinFN     ITQVEDTSAT LRFHMPSENG FTVTLEDEDS QPRTINLPQY VTYYQLEHLK PSTQYTMSLA NGDSTVDIDS LQFLTKPSPA TDFTAQEISP

       2881
HsaFN1    ---------- ---------- ---------- ---------- ---------- ---------- ---------- ---------- ----------
MonFN1    ---------- ---------- ---------- ---------- ---------- ---------- ---------- ---------- ----------
GalFN1    ---------- ---------- ---------- ---------- ---------- ---------- ---------- ---------- ----------
AnoFN1    ---------- ---------- ---------- ---------- ---------- ---------- ---------- ---------- ----------
XenFN1    ---------- ---------- ---------- ---------- ---------- ---------- ---------- ---------- ----------
DanFN1a   ---------- ---------- ---------- ---------- ---------- ---------- ---------- ---------- ----------
DanFN1b   ---------- ---------- ---------- ---------- ---------- ---------- ---------- ---------- ----------
PetFN1    ---------- ---------- ---------- ---------- ---------- ---------- ---------- ---------- ----------
PetFN2    ---------- ---------- ---------- ---------- ---------- ---------- ---------- ---------- ----------
CsaFN     DSFRLNWIQP ---------- ---------- ---------- ---------- ---------- ---------- ---------- ----------
CinFN     NGFTLTWSPP QGSINGYRLK YRPVSGNVDG EWNEVRIRPS DTEYTTTNLI PSTLYDVTIT PTYANPNAHR YSELGIPSSI QVTTIPLEVS

       2971
HsaFN1    ---------- ---------- ---------- ---------- ---------- ---------- ---------- ---------- ----------
MonFN1    ---------- ---------- ---------- ---------- ---------- ---------- ---------- ---------- ----------
GalFN1    ---------- ---------- ---------- ---------- ---------- ---------- ---------- ---------- ----------
AnoFN1    ---------- ---------- ---------- ---------- ---------- ---------- ---------- ---------- ----------
XenFN1    ---------- ---------- ---------- ---------- ---------- ---------- ---------- ---------- ----------
DanFN1a   ---------- ---------- ---------- ---------- ---------- ---------- ---------- ---------- ----------
DanFN1b   ---------- ---------- ---------- ---------- ---------- ---------- ---------- ---------- ----------
PetFN1    ---------- ---------- ---------- ---------- ---------- ---------- ---------- ---------- ----------
PetFN2    ---------- ---------- ---------- ---------- ---------- ---------- ---------- ---------- ----------
CsaFN     ---------- ---------- ---------- ---------- ---------- ---------- ---------- ---------- ----------
CinFN     TTPRTSPAPR PPSNVEFSHV TPVSVHASWT PSPDRDLDLY SVVLKDENGS TVVSGNLRNS FAIFRDLTPR SVYHLFVYAV RRDLQSVPAN

       3061
HsaFN1    ---------P TDLRFTNIGP DTMRVTW-AP --PPSIDLTN FLVRYSPVKN EEDVAELSIS PSDNAVVLTN LLPGTEYVVS VSSVYEQHES
MonFN1    ---------P TDLRFTNVGP DTMRVTW-AP --PSSIELTN FLVRYSPVKN EEDVAELSIS PSDNMVVLTN LLPGTEYLVS VSSVYEQHES
GalFN1    ---------P TDLRFTNVGP DTMRVTW-TA --PTSIVLSS FLVRYSPVKK EEDVAELTIS PSDNVVVLTN LLPGTEYLVR VYSVAEQHES
AnoFN1    ---------P TDLRFTNVGP DTIRVTW-TP --PASIELTS FLVRFSPVKN DEDVTELSIS PSDNAVVLTN LLPGTVYLVR VYSVYEQHES
XenFN1    ---------P TNLRFTNVGP DTMRVTW-SP --PTSIELSS FLVRYSPVKK PDDVTELSLS PSTNMVVLSN LLPFTEYLVS VHSCGPHKEF
DanFN1a   ---------P YGLSFGEVTA DTMLVTWKAP QVPKSSDINQ YIIRYHPVDE DDETTERTVE GSENFVVLRH LVPNTEYLVS VICVYEGREG
DanFN1b   ---------P TNLYISEVGA DSMHVSW-TA PSVQPSEISR FVIRYHPTNN DDDTQEVNVG GGTTSFVLQN LLPNTEYLVK VVCVYDDRES
PetFN1    ---------P TDLRFTQVEL DSFRVSW-SA --PPISTTES FLIRYSPLRH EDLLSEVSTT PDELEVILTG LLPGTVYGVS VHTVIRGHES
PetFN2    ---------P SDLRFSHVGA ESMHVSW-AP A-PPPASPTH YVVHYSPLYS SAEPREVVVA GDATSVDLGG LHPGLEYRVK VHAADGDKLS
CsaFN     ----AGINQP ERVSITNVGD SSFDVTW-LA EEPNNPLLTG YEVSVNELDS STEAQTSIFP ANINQ----- ---------- ----------
CinFN     ASVFTGQNHP ERVYITNIDD TSFDVAW-VP ERPTNPLLSG YEVSVKKQDS SSPAKSNFFP SNINQAVIQD LEPGTIYEVF VKSVINQVNT
```

```
           3151
HsaFN1     TPLRGRQKTG LDSPTGIDFS DITANSFTVH WIAP------ ---------- ---------- ---------- ---------- ----------
MonFN1     IPLKGIQKSG LDSPTGIDFS DITANSFTVH WMSP------ ---------- ---------- ---------- ---------- ----------
GalFN1     APLSGIQKTG LDSPTGLDFS DITANSFTVH WIAP------ ---------- ---------- ---------- ---------- ----------
AnoFN1     KPLSGEQKTG LDSPTGLDFS ETTAHSFTVH WMAP------ ---------- ---------- ---------- ---------- ----------
XenFN1     CIYSDLNTIY LDSPTGIDFS EITPNSFTVH WIAP------ ---------- ---------- ---------- ---------- ----------
DanFN1a    SPAIGTQRTV FDAPVGLQFS DVGTTSFTVR WQAP------ ---------- ---------- ---------- ---------- ----------
DanFN1b    EPVTGIQKTK LDSPTNLDFS DVSTNSFTVH WMAP------ ---------- ---------- ---------- ---------- ----------
PetFN1     VGLSGTQGTE IDAPTDMEFL DVTDSTFTVY WTPS------ ---------- ---------- ---------- ---------- ----------
PetFN2     AGASGTHKTA IDSPTQLEFL DVTDSSLTVA WQPP------ ---------- ---------- ---------- ---------- ----------
CsaFN      ---------- --ASQGLSF- ---TKKYY-- ---------- ---------- ---------- ---------- ---------- ----------
CinFN      DVINKQVSTL LPAPRNLRFE DVTDNSFYVT WDHPSLHIRD YVVEYQPIDS PDKLRLQVNN NNRIQLQNLQ PDSGYLISVY SLNYRRSSP

           3241
HsaFN1     ---------- ---------- ---------- ---------- ---------- ---------- ---------- ---------- ----------
MonFN1     ---------- ---------- ---------- ---------- ---------- ---------- ---------- ---------- ----------
GalFN1     ---------- ---------- ---------- ---------- ---------- ---------- ---------- ---------- ----------
AnoFN1     ---------- ---------- ---------- ---------- ---------- ---------- ---------- ---------- ----------
XenFN1     ---------- ---------- ---------- ---------- ---------- ---------- ---------- ---------- ----------
DanFN1a    ---------- ---------- ---------- ---------- ---------- ---------- ---------- ---------- ----------
DanFN1b    ---------- ---------- ---------- ---------- ---------- ---------- ---------- ---------- ----------
PetFN1     ---------- ---------- ---------- ---------- ---------- ---------- ---------- ---------- ----------
PetFN2     ---------- ---------- ---------- ---------- ---------- ---------- ---------- ---------- ----------
CsaFN      ---------- ---------- ---------- ---------- ---------- ---------- ---------- ---------- ----------
CinFN      VVGRQSTSSF SDLVSPANLR VDNVDTDIMR VNWEPSEHFT YTVVVTNPYG QVVSRQTFNS SRNFVDIDDL EPGILYKVQL RILTPTNRLA

           3331
HsaFN1     ---------- ---------- ---------- -------RAT ITGYRIRHHP EHFS-GRPRE DRVPHSRNSI TLTNLTPGTE YVVSIVAL--
MonFN1     ---------- ---------- ---------- -------RAP NTGYRIRHHP EHGV-GRPRE DRVPPSRNSI TLTNLNPGTE YVVSIISV--
GalFN1     ---------- ---------- ---------- -------RAT ITGYKIRHHP EHGV-GRPKE DRVPPSRNSI TLTNLLPGTE YVVSIIAV--
AnoFN1     ---------- ---------- ---------- -------RAP INGYKIRHYP EQGG-SRPKE HRIAPSRNSI TLTDLIPGSE YVVSISAF--
XenFN1     ---------- ---------- ---------- -------RAP ITGYRIRYQL ESGA-GRPKE ERVPPSRNSI TLTHLIPGSE YIVSIIAF--
DanFN1a    ---------- ---------- ---------- -------RAI ISGYRIRYQM TSG--GRAKE ERLPPSRSHF TLTGLTPETE YSISVYAV--
DanFN1b    ---------- ---------- ---------- -------RAV ITGYRLRYQP TSG--GRAKD ERLPPTRNYF TLVNLAPETE YTVHIYAV--
PetFN1     ---------- ---------- ---------- -------NAP VSGYRVAYGP QLL--GHVAE DSVPPERTSI VLASLQAGTE YTVSVYAIGR
PetFN2     ---------- ---------- ---------- -------RAA ITGYRVRYGP EGGAPGYANE RLVTGDRTQL ELHDLEPGTD YVVRVISV--
CsaFN      ---------- ---------- ---------- ---------- LKGYRLKYKA DDE--SVYHT LDLSSAQRSY TIPNLNPQTT YSIQLSAHVN
CinFN      GTEQVFQQTK PEAPRNVGLV SSNPRRIVMN WRPNRFRNPE ISGYKLTYET DDE--EIPHE VELGPRQHSY TINDLNPSTV YRITLQSIVN

           3421
HsaFN1     NGREESPLLI GQQSTVSDVP RDLEVVAATP TS-------- ---------L LISWDAPAVT VRYYRITYGE TGGNSP---V QEFTVPGSKS
MonFN1     NGRDESAPLV GQQSTVSDVP RDLEVIATTP TS-------- ---------L LISWDAPAVT VRYYRITYGE TGGSSP---V QEFTVPGTKS
GalFN1     NGREESVPLV GQQTTVSDVP RDLEVNPTSP TS-------- ---------L EISWDAPAVT VRYYRITYGE TGGSSP---V QEFTVPGTMS
AnoFN1     NGKEESLPLI GHQSTVSDVP RDLHVTATSP SS-------- ---------L VISWEPPAVT VRYYRITYGE TGSSGP---V QEFTVPGHMS
XenFN1     NGQQESLPLS GQQATVSDVP TDLEVTSSSP NT-------- ---------L TISWEAPSVN VRYYRITHSQ TGGHGP---E KEFTVPGTSN
DanFN1a    SGSRDSLPLT GTQSTISDAP TDLEVISSTP TS-------- ---------I TVRWDAPSVT VRYYRITHGE SGGSDA---P LEFMVPGSQS
DanFN1b    SSNIESLPLT GTQATVSDAP TDLAVTSSTP TS-------- ---------I TISWDAPAVT VRYYKITHGE TGERDA---P REFTVPGTQS
PetFN1     DVHRESEPLV GSQATNSNGP TDLVITRTSP TT-------- ---------F SVSWTPPPIS VRYYRLRGTH GGG-GARAVT ADHGVPVSRS
PetFN2     DGREESLPLM G-QTRTEGRP RRLEVAGESP SS-------- ---------L TLTWYQPSAP VDHYTVTCTP LSGREE---V RELLVPGSSS
CsaFN      NTLSEPVSIT A-RTQP---- ---------- ---------- ---------V SVGWRSVQGP VQYYRVIYVR RSG-GN---T TQISVPGRVN
CinFN      DTLSEPITTT V-LTQPEEAT DRMLTTTTTP NTNEALQGGI RTRKLNDGNY SIGWRSVSGP VEYYRVIYVR RSG-SN---A TQLSVPAHVN

           3511
HsaFN1     T-ATISGLKP GV--DYTITV YAVTGRGD-- -SPASSKPIS INYRTE---I DKPSQMQVTD VQDNSISVKW LPSSSPVTGY RVTTTPKNGP
MonFN1     T-ATISGLKP GV--DYTITV YAVTGRGD-- -SPASSKPVT IAYRTE---I DKPSQMQVTD VQDNSISVRW LPSSSPVTGY RVTTAPKNGS
GalFN1     R-ATITGLKP GV--DYTITV YAVTGRGD-- -SPASSKPVT VTYKTE---I DTPSQMQVTD VQDNSISIRW LPSSSPVTGY RVTAVPKKGH
AnoFN1     S-ATIPGLKP GA--DYTITV YAVTGRGD-- -SPASSKPVT VNYRLE---I DTPSQLQVTD VQDSSIRVRW QPSSSPVTGY RVTAAPKNGH
XenFN1     T-ATIRGLNP GV--SYTITV YAVTGRGD-- -SPASSKPLT IVHMTD---V DQPTDMAVTD IQDHSIHVKW SPPPGPVTGY RVTSVPKSGQ
DanFN1a    T-ATIEDLRP GT--DYTITV YAVTGRGD-- -SPASSTPIH VKHRTD---I DSPSQMEVTD VKDNTITVRW TPAAGPISRY RVNATPLTGE
DanFN1b    S-ATIQGLRP DT--DYTITL YAVTGRGD-- -SPASSTPVI ITHRTAGGSI ASPSDLDVTD IQDNTLTVRW TPARGPITGY RVTGTPKGGQ
PetFN1     EFAIFEKYLP PRPSPMNLFV FATFDRGA-- ----STARTA PAPGTE---V DTPSGLEVME VTDRTIRIRW APSKAPINGY RVTSIPKTGR
PetFN2     R-ATLQGLAT AQ--EYNVSI RATYGQQQQG GPMLSSQPVY IVHYLG---V DQPSDMQILD LTESSIRVRW NPCRAPILGY LLTCTPTTHP
CsaFN      E-ILLNNLNP AE--VYDVTI TAVLPNQE-- ---------I VVGNLE---I STSAESQPTE DSSRSAWIEW EQHPFMFTSY RITAIPVTG-
CinFN      E-IQLNKLIP GQ--EYDISI IAVYPNQE-- ---------V NIGNIA---V TTSGSDNVAP DSSRTALIEW DQQPLTFSSY RITATPVRG-

           3601
HsaFN1     GPTKTKTAGP DQTEMTIEGL QPTVEYVVSV YAQNPSGESQ PLVQTAVTNI D-RPKGLAFT DVDVDSIKIA WESPQ-GQVS RYRVTYSSPE
MonFN1     GPSKTKTTGP DQTEMTIEGL QPTVEYVVSV YAQNQNGESQ PLVQTAVTNI D-RPKGLAFT DVDVDSIKIA WESPQ-GQVS RYRVTYSSPE
GalFN1     GPTKTKNVPP DQTQVTIEGL QPTVEYMVSV YAQNQNGESL PLVETAVTNI D-RPKGLTFT EVDVDSIKIA WESPQ-GQVT RYRVTYSSPE
AnoFN1     GPTKTRVLSA EQTEVTIEGL QPTVEYIVSV YAQSQNGESQ PLVETAVTKI D-RPKGLTFT EVDVDSIKIA WESPQ-GQVS RYRVTYSTPE
XenFN1     GETFSQVVSP DQTEVTIVGL QPAVEYVVSI YSQGENGESE PLVETAVTNI D-HPKGLTFT DVGVDSIRLA WEVPD-GQVT RYRVTYSSPE
DanFN1a    GPVLHAETTS DHTEITFSGL MPAVEYSIKV YALGQDGESL PLVENVVTTV D-KPKDLSFT DVESSSMRIS WDSPD-GVVS SYRVLYYSPE
DanFN1b    GPTFSELVGP ERTEMTIHGL VPTVEYTINV VALSQEGEST PVVQKATTTN EQKPKDLTFT DVDSTSMLVK WNVPRVPGVT SYRVLYSSPE
PetFN1     GTQDTRELPS GRTDVTIDGL LPTLEYAVNV YALSDHGESL PASKNVVTGI D-APKGLTFT DVDVDSMSIS WDSPE-GTVT GYRVLYESLD
PetFN2     AGPSTFAIPP GETDVTITKL VPTVEYVISV CARRHNQDSE PTTLTFTTKV D-SPRGLEFL QVAETWVLVQ WEPPE-GRVS SYRITYESSN
CsaFN      GQTHRWEVGA GESNARLSDL EPGTLYNILV EGFTNGQYVT VADIQRRTDI D-PPSNLRII KSNTNVIGLH WNPPK-SNIT GYSLTYQLDG
CinFN      GQTHTWNIRS SETSAHLSDL EPGTLYHIVV EGYLDGQYVT VADIEKTTDI D-SPSNLEVT KINTNLIGIE WSPPN-SQIS GYSLTYQLDG

           3691
HsaFN1     DGIHELF-PA PDGEEDTAEL QGLRPGSEYT VSVVALHDDM ESQPLIGTQS T--------A IPAPTDLKFT QVTPTSLSAQ WTPPN-VQLT
MonFN1     DGIHELF-PA PDGEEDTAEL QGLRPGSEYT VSVVALHDDM ESQPLIGTQS T--------A IPSPTNLKFS QVSPTSLTAQ WVAPN-VQLT
GalFN1     DGIHELL-PA PGGEEDTAEL HGLRPGSEYT INIVAIYDDM ESLPLTGTQS T--------A IPPPTNLKFT QVTPTSLTVN WNAPN-VRLT
AnoFN1     DGIQELF-PA PDGEDDTAAL HGLRPGTEYT VRIIALHDGL ESLPLVGTQS T--------A IPPPTNLRFG QITPTSVTIT WTAPN-VRLT
XenFN1     EGVKELF-PA PEGDDDTAEL HGLRPGTEYT VSIVALHDDM ESKPMIGIQS T--------A IPAPTNLQFS QVTPSGFSLS WHAPT-IHLS
DanFN1a    EGERELF-PA PHGDDESAVL HGLRPGTEYT VKVIALHDQS PSTALVGTQT T--------G IPGPTRLQFS QVGPTSFTVS WSSSD-ASLT
DanFN1b    EGERE-YQPA LSGRDNSVLL QGLNPGTMYI IKVIPMKGRN PLQTLEGIQQ TIQEDTQRIA VPAPTDIQFF DISPSSFIVS WQAPN-SRLS
PetFN1     DGVPQTLSPA PDGDADTAVI SQLQPGSDYK VSVTALHHGT ESLPLMGTQA T--------A IPPPTALKLV QATSTSLSIQ WQPPS-AEVS
PetFN2     EGTRE-FPHT PRGDVEEANI TGLTPGLEYT VRVYATYGQD ESVPLTGCQV T--------A IPAPTDLQFH FVTASGLTVS WAAPVGVELT
CsaFN      RGTETAYIPP PRSTDVRKTL RRLQPGQRYF IRLYSRFGDV ESLPAQVWAE T--------L VPAPQRFRIT QATPNMITLS WVKPV-DIVT
CinFN      RGTETAYIPP PRVSDVRKTL RRLQPGQRYV IRLYAHLNDM ESLPAQVWAE T--------S VPSPQRFRIT QATPNLISIS WIKPS-HLIT
```

```
        3781
HsaFN1   GYRVRVTPKE KTGPMKEINL APDSSSVVVS GLMVATKYEV SVYALKDTLT SRPAQGVVTT LENVSPPRRA RVTDATETTI TISWRT-KTE
MonFN1   GYRVRVTPKE KTGPMKEINL SPDSTSVVVS GLMVATKYEV SVYALKDTLT SRPAQGVVTT LENVSPPRRA RVTDATETTI TINWRT-KTE
GalFN1   GYRVRVNPKE KTGPMKEINL SPDSTSAVVS GLMVATKYEV SVYALKDSLT SRPAQGVVTT LENVSPPRRA RVTDATETTI TITWRT-KTE
AnoFN1   GYRVRVNPKD KNGPMKEINV SPDSTSAVVS GLMVATKYEV TVYALKDSLT SRPAQGVFTT LENVSPPRRA RVTDVTERTI TITWRT-KTE
XenFN1   GYLVRVNPKE KTGPMKEVRL SPGVTATTVT GLMVATKYEV NVYALKDTLT SQPLQGLIST LDNVSPPRRP RVQDVTETTV TLSWRT-KTE
DanFN1a  GYRVAVSPKS KSGPTKEENI TPDSTEFHAT GLMPGTDYEV EVYGVKNSLT SPRVKGEITT PDNISPPRRV RISNVKDSSI TLTWRS-KTE
DanFN1b  GYRVVVTPRN QLATSKEMTI SPDSTQVLVP GLVVATPYNV HVYALRGSDS SPPLTGEITT ADNITPPRRV RISDVKDTSF TLAWRAVNNE
PetFN1   GFRVVVTPRE KSGPSRDHMV PADVNSFTLP SLMVATQYNV FVYALKNGHT SKPLQDVFTT LENISPPRKP RVVNVTETSI TVNWRA-KSD
PetFN2   GFRVVLMPRD RKGPTLERSQ SPKVTTTTIN GLLAQTEYEV SVYALKNYLA SKSLQAFQKT QQSVMPPGRL RALSVTGSTV LVAWRS-RAV
CsaFN    HYTLRVIGPN GFDVLQNLRL GPDTNTFTVH RLQPGTRYHI TLAAWIGSLS SKPEYKSKVT PLTV--PVVK SSGEPTGEGF LLQWKG-KSR
CinFN    HYTLRVIGPD GFDVIHNLRL DDDVNSYTIR DLQPGTRYHI NLAAWIGPLS SQPEYKSKIT PETV--PDVK TKGELTDQGF LLQWKS-KSR

        3871
HsaFN1   TITGFQVDAV PANGQ-TPIQ RTIKPDVRSY TITGLQPGTD YKIYLYTLND NARSSPVVID ASTAIDA--- ----PSNLRF LATTPNSLLV
MonFN1   TITGFQVDAI PSNGQ-SPIQ RTISPEVRSY TITGLQPGTD YKIHLYTLND NARSSPVIID ASTAIDA--- ----PSNLRF LTTTPNSLLA
GalFN1   TITGFQIDAI PAASGQNPIQ RTISPDVRTY TITGLQPGND YKIYLYTLNE NARSSPVVID ASTAIDA--- ----PSNLRF LTTTTNSLLA
AnoFN1   TITGFQIDAI PASGQ-NPVQ RTIGPDVRTY TITGLQPGTD YKIYIYTLND NARSSPVVID ASTAIDA--- ----PSNLRF LTTTTNSLLL
XenFN1   TITGFQIDAI PAGGQ-NPIR RTVDADLRSF TITGLQPGTD YKIYLYTLND NARSSPVIVD VTTAVDS--- ----PSNLRF LTTTSNSLLF
DanFN1a  AISGFLVEAT PTMGGHNPIQ RTIEPDSRTY TIAGLEPGTN YKINIYTLNG NSRSEPFTLT ATTAKPVISP ----PTNLHF TSLASTSISF
DanFN1b  PMTGFLIEAT PKNGGYPTFR QTIPSDRRTY VVTGLHPGVM YVVNMYTVNG NMRSPPFTMT VNTARPAVQS ----PTNLQF TSLTPNSISF
PetFN1   TLSGFLISVV PSSTA-PSVE KTLSPDARQF TIADLLPGIH YTISIYAVSG NTRSQPATVT ATTALRSLDA ----PKDLRF DSASQDSITF
PetFN2   RLSAFLLIAT PLSNEGLSAE RLLSPLLRAC TITGV----- ---------- -----PPLSL ARSHMYTHTF --NGAHSHAL AHVDV-CVGA
CsaFN    SVDGFKIVVE PQDGS-AEFE TTVDRFTKRY QIENLLNNVE YIVKIFPVSG RIVGEPSIVR IPAHRYSTT- ----SWRLQV THLNANSVRI
CinFN    SVDGFKIVVE PQDGS-AGFE TTVDRFTKSY ELENLLSNVA YIVKVYPVSG HIIGEPSVVH IPASTASNVA LNHLQWRLQV TSLGTDSIAI

        3961
HsaFN1   SWQPPRARIT GYIIK---Y- --EKPGSPPR EVVPRPRPGV TEATITGLEP GTEYTIYVIA LKNNQKSEPL IGRKKTDELP QLVTLPH--P
MonFN1   SWQPPRARIT GYIIK---Y- --EKPGLPPR EVVPRPRPGV TEATITGLEP GTEYIIYVIA LKNNQKSEPL IGRKKTG--- ----------
GalFN1   SWQPPRAKIT GYIIR---Y- --DKPGSPAK ELLPRPRPGT TEATITGLEP GTEYTIYIIA VKNNQKSEPL VGRKRTD--- -DLPTLITGP
AnoFN1   TWHPPRAKIT GYIIK---Y- --ERPGGPAR ELLPRLRPGM TEATITGLEP GTEYTIYIIA VKNNQKSEPL IGRKKTDE-- --HPQLV--T
XenFN1   TWQPPRARIT GYIIR---Y- --EKAGGLLK EHLPRLQAGT TESTITNLEP GTEYIVYIIA VRNNQKSEPL VGRKRTDELP -RLVTLP--H
DanFN1a  TWEPPRSTIT GYYVT---Y- --EEAGGIPK ELTPRPQAAR TFASISGLIP GTEYIIKIVA LNGAQRSTPL IGKARTQLEI EQLPPQL--P
DanFN1b  TWQAPPTHIT GYYIT---Y- --EESGGSPR ELFPRNNVGQ NYATIAGLKP GTEYIIKIIA LLNNQRSAPL VGTATTQLIS -GLPSL---P
PetFN1   SWQPPRAPIM SYVVT---YS --SEVG-SLN RLTPAPGPRD TSATITNLQP STRYTIGIYA VQNSMRSAQL TGKHRTEQEL -QLPQLA--T
PetFN2   QFHVHYLTTI YLLLRQPPFT LIEMYS-TSR VILKHPQPGI FQTPITRLSP GVTYSISIYA MREGSRSQP- -TSITVT--- ---TSKA--P
CsaFN    IWNNPWPDVT EYILE---YL LVNEND-FYH LVERQPMVGD NSATINSLQS GKTYTIKLTA VRDSTDSGG- -ESIRNTLLL -NVTTLI--P
CinFN    SWNNPWHDVV EYSLQ---YK QIDESDEYYR SVVPQPQIGD NHATISHLQP GESYTIKLAV RRDSQSDG-- ---LMNTLLL -NVSTSI--P

        4051
HsaFN1   NLHGPEILDV PSTVQKTPFV THPGYDTGNG IQLPGTSGQQ PSVGQQMIFE EHGFRRTTP- PTTATPIRHR PRPYPPNVGE EIQIGHIPRE
MonFN1   ---------- ---------- -------QEA LSQTTISW-T PFQESSEY-- IISCHPIG-- ---------- ---------- ----------
GalFN1   HPNQPDMLDV PSVDEGTPYL TN-----NRP GSRRQPPNVD EAIEIPGYQV PIIVVPSYP- ---------- ---------- ----------
AnoFN1   LPHHSQGTSG QQPIV----- -------TNP GQVIVEVYGS RTPTTAAT-- PSRHRPGEE- ---------- ---------- --------SG
XenFN1   PGKGPEILDV PTDEENTPHI TQIKLDNGNG IQLPGSNGQQ PSSGNEGQ-- LIEEHGFRS- ----PLAPTT AVPVRPGKFT PGHYPQERVD
DanFN1a  NPSRPHLEVL DVPEDNDNF- -------NNH VHVLGPNRHN TLGQQGQHI- YTEYQSFNL- ------GNNG QQPHHPNHGE PLVYIPLPGA
DanFN1b  SHGGRDKLDV PEP------- -------DNR INTVGPTGHD STDAHGQHVE YTEYNNQPKQ PNIGYQPQNR QPISQPNQPR PQPYVPQVGE
PetFN1   RPPNFPARYI PPFLDIPTS- -------SQH SSTVLERDPN SESRSTRY-- YTRQRSLNNR QNGRPLSGQR PVGDFLTALT PSHSPDRSAA
PetFN2   SPQR------ ---------- -------RSS GNI------- ---HSKDF-- LTAQSSL-P- ---------- ---------- ----------
CsaFN    TMSNIRLHHL ---------- -------NND MSMVTFSWEA PPMHVSSF-- VIKYREIDS- ---------- ---------- ----------
CinFN    PPSNIQMQR- ---------- -------SSD GSLV-FTWQS PTSYASNY-- IVKYREVYP- ---------- ---------- ----------

        4141
HsaFN1   DVDYHLYP-- ---------- ---------H GPGLNPNAST GQEALSQTTI SWAPFQDTSE YIISCHPVGT -DEEPLQFRV PG--TSTSAT
MonFN1   -IDEEPL--- ---------- ---------- ---------- ---------- ---------- ---------- ------QFRV PG--TSASAT
GalFN1   -HSREPRR-- ---------- ---------- ------NDTT GQEALSQTTI SWRPLLESTE YIISCQPVSQ -DEDTLQFRV PG--TSSSAT
AnoFN1   VVDRHPGRIP EVDRPS---- --YPHTLL-- ---PKNNRTR PQEALSETTI AWTPYLESSE YIISCQPVSS -EEEPTEIKV PG--TYSSAT
XenFN1   IELDTEFP-- ---------VQ RGDLDGPYPH GLGPQLNDSG VQEVASHTTI SWRPELETTE YIISCHPIEH -DEAPLQFRV PG--TSSSAT
DanFN1a  DGQRVPVV-- ---------K VNEGQEAGFP FGGLFNETNL PQESQTQTTI VWQPVPHTSE YVVWCDPITE INEKSFQMRL PG--TSTSAT
DanFN1b  TLVYVPQVGP DGGRVPQIIQ VSERPGDGHT FGFPEDRTGR PQEAQTQTTI SWQPFQQSSA YLVSCQPITH PDEKMFQMRV PG--TSTTAT
PetFN1   GFTPSEVVFM DRGLVP---- -----IKT-- ---EDRILGP QEAAKTLTTI SWSPQPQASE YLLSCYPLSG -EGETTQIRV PA--GTSSAT
PetFN2   -MK----I-- ---------- ---------- ---------- ---------- ---------- ---------- ------TLQD PGVREGNGVR
CsaFN    -ITQHDYI-- ---------- ---------- ---------- ---------- ---------- ---------- ------TIEV PG--TNTTVN
CinFN    -FLQHEYV-- ---------- ---------- ---------- ---------- ---------- ---------- ------VMEL SG--SDTSVT

        4231
HsaFN1   LTGLTRGATY NVIVEALKDQ QRHKVREEVV TVGNSVNEGL NQP---TDDS CFDPYTVSHY AVGDEWERMS ESG-FKLLCQ CLGFGSG---
MonFN1   LTGLTRGATY NIIVEALKNQ RRHKVREEMV TVGNSVNEGL NQP---TDDT CFDPFTVTHY SIGEEWERLS DSG-FKLWCQ CLGFGSG---
GalFN1   LTGLTRGATY NIIVEALKDH RRQKVLEEVV TVGNTVSEGL NQP---ADDT CYDTYTGSFY SIGEEWERLS ETG-FKLWCQ CLGFGSG---
AnoFN1   LTGLTRGATY NIIVEAIKNR RRHKVLEEVV TVGNTVSERL NQP---TDDS CYDTFTGSFY SLGEEWERMS ETG-FKLWCQ CLGLGSG---
XenFN1   LNGLTQGATY NIIVEAQKGS DKHKVFEKRV TVGSPGS--P EGVLQPAEDT CYDTFNGAHY SVGQEWERMS ESG-FRLWCK CLGYGSG---
DanFN1a  LIGLTSGASY NVLVESVNGD QKQKVLQDVV TVGNSVP--G AGVIPTGRDV CYDSFTATYH EVNTEWERMS ETG-FKLWCK CLGLGSG---
DanFN1b  LIGLTSGASY NVIVEALKGA LKHKILEEVV TAGNTVP--G DAT--SSKDS CYDTFTATHH DVGAEWERMS DTG-FKLWCR CLGLGSG---
PetFN1   LAGLADGTQY SVVVEALQGT QRYRVHEEVV TARAVSS--P HEGVESDEDL CLDTATSIYY SVGQEWERLS ENG-FMLYCR CLGFGNG---
PetFN2   RPSSN-GATW EGGFE----- -----REEFL GAASPTF--L RDG---RSDH VGAAPGQLHT TV--TW--RP SGGSTEHHVT CLPIGDSSPV
CsaFN    IPDMNPGKSY EFQFYSKLGN L----RSDVV KVTAEIP--F RQT---PERH CQDPDTHEIF AIGQHW--IS KAGEFLLNCT CHGPST----
CinFN    MPFLQEGKRY EIVFYSKRGE F----RSDSV VVNVDVP--V SQQ---VRNY CTDPDTGATY AVGQNW--LS KAGLFLMNCT CRPSLT----

        4321
HsaFN1   -HFRCDSSRW CHD-----NG VNYKIGEKWD RQGENGQMMS CTCLGNGKGE FKCDPHEAT- ---CYDD--G KTYHVGEQW- QKEYLGAICS
MonFN1   -HFRCDSSKW CHD-----NG VNYKIGDKWD RQGENGQMMS CTCLGNGKGE FKCDPHEAT- ---CYDD--G KTYHVGEQW- QKEYLGAICS
GalFN1   -HFRCDSSKW CHD-----NG VNYKIGEKWD RQGENGQMMS CTCLGNGKGE FKCEPHETT- ---CYDD--G KMYQVGEQW- QKEYFGAICS
AnoFN1   -HFRCDSSKW CHD-----NG VNYKIGEKWD RHGENGEMMS CTCLGNGKGE FKCEPHEAT- ---CYDE--G KMYHVGEQW- QKEYLGAICS
XenFN1   -HFRCDSSKW CHD-----NG VNHRVGEKWD RRGENGQMMS CTCLGNGKGE FKCEPHEAT- ---CYDE--G KMYNVGEQW- QKEYLGAICS
DanFN1a  -HFRCDSSKW CHD-----SG HNYRIGEKWD RRAENGHMMS CTCLGNGKGE FKCEPHEST- ---CYDE--G KLYQVGNQW- QKEYLGAICT
DanFN1b  -HFRCDSSKW CHD-----NG VNYKIGEKWD RRAENGHLMS CTCLGNGKGE FKCEPHDSI- ---CYDE--G KMYQIGNQW- QKEYLGAICT
PetFN1   -HYRCDSSRW CHD-----NG KNYREGETWD RPADNGQMLT CTCLGRGKGE YKCEPHEAS- ---CYDE--G KMYQAGDQW- RKLYRGAMCT
PetFN2   VKLTLPGGAE AATLRGLRPG AVYRVLVEA- MGLDGRNTLL DETLSLNNTG TTEGAGEARI LDGCLEGTTG TAYAPGEHWE QRAQNGLLLT
CsaFN    --FDCNTGEW CHT-----DN GVFNVSDTW- RQVNDENNKK EECVCNGRTG YTCFPRN--- ---CQDG--D NWFDEGEPW- ISAIDGYICN
CinFN    --VDCSSGEW CHT-----EY GVFNKTNTW- RQDNPENNKK EECVCNGSSG YTCYPRN--- ---CQDG--D RWYDEGEPW- TSDIDGYICN
```

```
           4411
HsaFN1     CTCFGGQRGW RCDNCRRP-- ---------- ---------- ---------- ----GGEPSP EGTTGQSYNQ YSQRYHQRTN TNVNCPIECF
MonFN1     CTCFGGQQGW RCDNCRRP-- ---------- ---------- ---------- ----GVETAP EGSAGHSYTQ FSQRYHQRTN TNVNCPIECF
GalFN1     CTCYGGQQGW RCDNCRRP-- ---------- ---------- ---------- ----SVEVAP EGSAGHTYPQ FTQRYHQATN TEEDH-YSDL
AnoFN1     CTCYGGQQGW RCDNCRRP-- ---------- ---------- ---------- ----GAGSSP E-SSPGHTSS LYTQRHQTTN TNVHCPIECF
XenFN1     CTCYGGQQGW RCDNCRRP-- ---------- ---------- ----GA---- -------VLP DGTAGHTVSQ FAQRYQQNYN L--NCPIECY
DanFN1a    CTCYGGQQGW RCENCRRP-- ---------- ---------- ---------- GAEVDADLIQ PPVRTDAFDR YRENALRKLN I--QCPIECL
DanFN1b    CTCYGGQQGW RCENCRRP-- ---------- ---------- ----GAEIN- ------ADLL RP-------- -VRLNNGQ-R VNIQCPIECL
PetFN1     CTCLGGQQGW RCEECRSP-- ---------- ---------- ----SEEDAG SSHTAAASHS SSASQGVFSQ YIQSVRRSAG T--NCAFNCR
PetFN2     CTCLGEGRGH VRCDSSK--- ---------- ---------- ---------- ---------- ---------- ---------- ---WC-----
CsaFN      CICPSDLHAP CRTTCRPQTT NQGPIKDLIF LVEGLPSIPK NITSGAENNS SDKVELLV-F EEDKSDGSHN YSDDFEREPG GGMNCPIQST
CinFN      CVCPSEHTQP CTSTCRPSIL GQ-------- ---------- ---------- ------DIGF ESKR------ ---------- ----------

           4501
HsaFN1     MPLDVQADRE DSRE--
MonFN1     MPLDVQADTE NSRDS-
GalFN1     E--------- ------
AnoFN1     YPLHLQSNTQ NRRGD-
XenFN1     L-PLQADIQH SEQTQK
DanFN1a    RPDLLADAHS PHD---
DanFN1b    RPELLADAVV NPKTQE
PetFN1     ---------- ------
PetFN2     ---------- ------
CsaFN      DIG------- ------
CinFN      ---------- ------
```
